# Identification of Biologically Essential Nodes via Determinative Power in Logical Models of Cellular Processes

**DOI:** 10.3389/fphys.2018.01185

**Published:** 2018-08-31

**Authors:** Trevor Pentzien, Bhanwar L. Puniya, Tomáš Helikar, Mihaela T. Matache

**Affiliations:** ^1^Department of Mathematics, University of Nebraska at Omaha, Omaha, NE, United States; ^2^Department of Biochemistry, University of Nebraska-Lincoln, Lincoln, NE, United States

**Keywords:** Boolean networks, signal transduction network, determinative power, mutual information, simulations, cell collective, gene essentiality, statistical analysis

## Abstract

A variety of biological networks can be modeled as logical or Boolean networks. However, a simplification of the reality to binary states of the nodes does not ease the difficulty of analyzing the dynamics of large, complex networks, such as signal transduction networks, due to the exponential dependence of the state space on the number of nodes. This paper considers a recently introduced method for finding a fairly small subnetwork, representing a collection of nodes that determine the states of most other nodes with a reasonable level of entropy. The subnetwork contains the most determinative nodes that yield the highest information gain. One of the goals of this paper is to propose an algorithm for finding a suitable subnetwork size. The information gain is quantified by the so-called determinative power of the nodes, which is obtained via the mutual information, a concept originating in information theory. We find the most determinative nodes for 36 network models available in the online database Cell Collective (http://cellcollective.org). We provide statistical information that indicates a weak correlation between the subnetwork size and other variables, such as network size, or maximum and average determinative power of nodes. We observe that the proportion represented by the subnetwork in comparison to the whole network shows a weak tendency to decrease for larger networks. The determinative power of nodes is weakly correlated to the number of outputs of a node, and it appears to be independent of other topological measures such as closeness or betweenness centrality. Once the subnetwork of the most determinative nodes is identified, we generate a biological function analysis of its nodes for some of the 36 networks. The analysis shows that a large fraction of the most determinative nodes are essential and involved in crucial biological functions. The biological pathway analysis of the most determinative nodes shows that they are involved in important disease pathways.

## 1. Introduction

Boolean networks have gained popularity as models for a variety of real networks where the node activity can be described by two states, 1 and 0, “ON and OFF”, “active and non-active,” and where each node is updated based on logical relationships with other nodes (e.g., Albert and Thakar, 2014; Abou-Jaoudé et al., 2016). Applications of such models include signal transduction in cells (e.g., Helikar et al., 2008; Conroy et al., 2014; Abou-Jaoudé et al., 2015; Mendéz and Mendoza, 2016), genetic regulatory networks as well as other biological processes (e.g., Kauffman, 1993; Klemm and Bornholdt, 2000; Shmulevich et al., 2002; Albert and Othmer, 2003; Shmulevich and Kauffman, 2004; Saadatpour et al., 2013).

However, even such a simplification of reality can pose challenges in assessing the dynamics of the network due to the exponential dependence of the state space on the number of nodes. One way to ease the computational burden is to reduce the network to a fairly small subset of nodes that can capture the dynamics of the whole network to a large extent. Some approaches deal with the elimination of nodes that become part of an attractor in the long run, and may also consider removing nodes that are not inputs to any other nodes (Bilke and Sjunnesson, 2001; Richardson, 2004). One can also consider merging or collapsing mediator nodes with one input and one output (Saadatpour et al., 2013). Yet, other approaches consider eliminating irrelevant nodes that are frozen at the same value on every attractor, together with nodes whose outputs go only to irrelevant nodes (Socolar and Kauffman, 2003; Kaufman et al., 2005; Kaufman and Drossel, 2006). In Veliz-Cuba (2011) the author uses a “steady-state approximation” by replacing variables in the Boolean functions governing the nodes' dynamics with their own Boolean expressions, thus reducing the network to a much smaller size that can be used to infer properties about the original network and to gain a better understanding of the role of network topology on the dynamics. In Naldi et al. (2009b) the authors introduce a general method for eliminating nodes sequentially by directly connecting the inputs of a removed node to its output nodes in a manner similar to Veliz-Cuba (2011). Of course, one needs to pay attention and possibly keep nodes that are or may become self-inputs upon elimination of other nodes. The order in which nodes are removed is also important. It is shown that stable states are preserved. In general, attractors may not be preserved. However, the method presented in Saadatpour et al. (2013) is shown to preserve attractors as well.

We consider a recently proposed method for identifying the most powerful nodes in a Boolean network (Heckel et al., 2013; Matache and Matache, 2016). This is done by finding the nodes with the highest determinative power. For a given node, the determinative power is obtained via a summation of all mutual information quantities over all nodes having the given node as a common input. The more powerful the node, the more the information gain provided by the knowledge of its state. The mutual information, as a basic concept in information theory, allows one to represent the reduction of the uncertainty or entropy of the state of a node due to the knowledge of any of its inputs. The entropy has been used in the literature to find the average mutual information of a random Boolean model of regulatory network as a way to quantify the efficiency of information propagation through the entire network (Ribeiro et al., 2008). On the other hand, the entropy of the relevant components of the network, which are comprised of nodes that eventually influence each other's state, has been used as a measure of uncertainty of the future behavior of a random state of the network (Krawitz and Shmulevich, 2007a,b).

In Heckel et al. (2013) it is shown that the knowledge of the states of the most determinative nodes in the feedforward regulatory network of *E. coli* reduces the uncertainty of the overall network significantly. Similar results are observed in Matache and Matache (2016) for a model of general cell signal transduction. It is our goal to explore other models of biological processes obtained from the Cell Collective (http://cellcollective.org), to identify any similarities or differences with respect to previous observations, and to possibly identify any correlations with other network variables or trends in the observed network data. At the same time, we show that the majority of nodes with the most determinative power are essential. Cell Collective provides a variety of gene networks. Essential genes are those genes of an organism that are thought to be critical for its survival and are involved in crucial biological functions.

In section 2, we provide the basic mathematical framework and definitions. We present the algorithm for finding a suitable subnetwork size in section 3. In section 4 we describe the networks under consideration and we provide the results of our simulations paired with a statistical analysis of the data. Then we focus on the analysis of the biological relevance of the most determinative nodes. We provide a discussion of the results in section 5. Conclusions and further directions of research are in section 6.

## 2. Determinative power

In this section, we provide the main concepts leading to the determinative power of nodes in a Boolean network.

**DEFINITION 1**. *Let* Ω^*n*^ = {0, 1}^*n*^*. A Boolean network (BN) is modelled as a set* [*n*]: = {1, 2, …, *n*} *of n nodes, each node being ON (in state* 1*) or OFF (in state* 0*). Then any* ω∈Ω^*n*^ *is a possible state of the network. Each node i*∈[*n*] *has an associated Boolean function* $f_{i}:\Omega^{n}\to \Omega$ *that governs the dynamics of the node*.

We are usually interested in how the network evolves by iterating the map *F* = (*f*_1_, *f*_2_, …, *f*_*n*_) a large number of times.

In this paper, a subnetwork refers to a subset of nodes of the network. One recent approach for finding subnetworks whose nodes determine the states of most other nodes with a reasonable level of entropy focused on the nodes with the most determinative power (DP) (Heckel et al., 2013; Matache and Matache, 2016). The DP is obtained via concepts from information theory. We recall the main definitions and concepts from Cover and Thomas (2006) and Heckel et al. (2013). These include the notion of entropy of random variables, which is a measure of uncertainty, and the mutual information, which is a measure of dependence between two random variables and is defined in terms of the entropy.

**DEFINITION 2**. *Let X and Y be discrete random variables. The (Shannon) entropy of X is defined as*

$$H\left(X\right)=-\underset{x}{\sum }p_{x}\log_{2}p_{x}=-E\left[\log_{2}P\left(X\right)\right]$$

*where x are the values of the random variable X*, *p*_*x*_ = *P*(*X* = *x*)*, and E*[log_2_*P*(*X*)] *is the expected value of the random variable* log_2_*P*(*X*)*. In binary this reduces to the function*

$$\begin{array}{l} h(p)=-p\log_{2}(p)-(1-p)\log_{2}(1-p),\\ \;p=P(X=1),\;h(0)=h(1)=0. \end{array}$$

The conditional entropy of Y conditional on the knowledge of X is

$$H\left(Y|X\right)=-E\left[\log_{2}P\left(Y|X\right)\right].$$

*The mutual information (MI) is the reduction of uncertainty of the random variable Y due to the knowledge of X. That is*

MI(Y;X)=H(Y)-H(Y|X).

In principle, the mutual information is a measure of the “gain of information,” or the determinative power (DP) of *X* over *Y*. The authors of Heckel et al. (2013) use the MI to construct the DP of a node *j* over the states of a Boolean network, namely

(1) $$\begin{array}{r} DP\left(j\right)=\overset{n}{\underset{i=1}{\sum }}MI\left(f_{i}\left(X\right);X_{j}\right) \end{array}$$

which represents a summation of all “information gains” obtained from node *j* over its outputs (i.e., nodes *i* that have *j* as an input). Here, the states of the nodes are labeled *X*_1_, *X*_2_, …, *X*_*n*_, and *X* = (*X*_1_, *X*_2_, …, *X*_*n*_) represents the state of the network. The notation *f*_*i*_(*X*) represents the random variable that describes the dynamical rule of node *i*. Not all variables *X*_1_, *X*_2_, …, *X*_*n*_ are relevant for the computation of *f*_*i*_(*X*) since the actual number of inputs may differ from one node to another. The authors identify the nodes with the largest determinative power in a feedforward *E. coli* network, with the goal of finding a subnetwork whose knowledge can provide sufficient information about the entire network; in other words the entropy of the network conditional on the knowledge of that subnetwork is small enough. They show that in the *E. coli* network, one could consider a subnetwork consisting of less than half of the nodes, and that for larger subnetworks, the entropy does not improve significantly once an approximate (threshold) subnetwork size is reached. Similar results have been found in Matache and Matache (2016) for a signal transduction model in fibroblast cells, paired with a mathematical generalization of some of the results in Heckel et al. (2013) under more relaxed assumptions. Our goal is to use a similar approach for other networks to identify if this type of behavior is typical or not. In the next section, we describe the networks under consideration and then we present the algorithm for finding a suitable subnetwork size. However, before we do that, let us provide an example illustrating the computation of DP according to formula (1). The mutual information terms in (1) are obtained using a formula derived in Matache and Matache (2016). We combine Theorem 1 and Proposition 4 of Matache and Matache (2016) in a suitable way to provide a brief explanation of how the formula is obtained.

The mutual information formula *MI*(*f*_*i*_(*X*);*X*_*j*_) can be written as

$$\begin{array}{r} MI\left(f_{i}\left(X\right);X_{j}\right)\\ \;=h\left(\underset{x\in \text{supp}f_{i}}{\sum }p_{x}\right)-P\left(X_{j}=1\right)h\left(\underset{x\in \text{supp}f_{i}}{\sum }P\left(X=x|X_{j}=1\right)\right) \end{array}$$

(2) $$\begin{array}{r} \;-P\left(X_{j}=0\right)h\left(\underset{x\in \text{supp}f_{i}}{\sum }P\left(X=x|X_{j}=0\right)\right) \end{array}$$

where supp*f*_*i*_ = {*x*:*f*_*i*_(*x*) = 1} is the support of the function *f*_*i*_, and *P*(*X* = *x*|*X*_*j*_ = *x*_*j*_) is the conditional probability of *X* = *x* given *X*_*j*_ = *x*_*j*_.

The formula follows directly from the definition of the mutual information

(3) $$\begin{array}{r} MI\left(f_{i}\left(X\right);X_{j}\right)=H\left(f_{i}\left(X\right)\right)-H\left(f_{i}\left(X\right)|X_{j}\right). \end{array}$$

Observe that

(4) $$\begin{array}{l} H(f_{i}(X))=h(P(f_{i}(X)=1))\\ \;=h(E[f_{i}(X)])\\ \;\begin{array}{cc} = & h(\underset{x\in {\left\{0,1\right\}}^{n}}{\sum^{\text{​}}}f_{i}(x)p_{x})=h(\underset{x\in suppf_{i}}{\sum^{\text{​}}}p_{x}) \end{array} \end{array}$$

where we use the known fact that for a (Bernoulli) random variable *B* with values 0 and 1, we have that *P*(*B* = 1) = *E*[*B*]. Similarly,

$$\begin{array}{l} H(f_{i}(X)|X_{j})=\underset{x_{j}\in \{0,1\}}{\sum^{\text{​}}}P(X_{j}=x_{j})H(f_{i}(X)|X_{j}=x_{j})\\ \;=\underset{x_{j}\in \{0,1\}}{\sum^{\text{​}}}P(X_{j}=x_{j})h(P(f_{i}(X)=1|X_{j}=x_{j})). \end{array}$$

On the other hand,

$$\begin{array}{l} P(f_{i}(X)=1|X_{j}=x_{j})=E[f_{i}(X)|X_{j}=x_{j}]\\ \;=\underset{x\in {\left\{0,1\right\}}^{n}}{\sum^{\text{​}}}f_{i}(x)P(X=x|X_{j}=x_{j})\\ \;=\underset{x\in suppf_{i}}{\sum^{\text{​}}}P(X=x|X_{j}=x_{j}). \end{array}$$

This implies

(5) $$\begin{array}{l} H(f_{i}(X)|X_{j})=\underset{x_{j}\in \{0,1\}}{\sum^{\text{​}}}P(X_{j}=x_{j})h(\underset{x\in suppf_{i}}{\sum^{\text{​}}}P(X=x|X_{j}=x_{j}))\\ \;=P(X_{j}=1)h(\underset{x\in suppf_{i}}{\sum^{\text{​}}}P(X=x|X_{j}=1))\\ \;\begin{array}{c} +P(X_{j}=0)h(\underset{x\in suppf_{i}}{\sum^{\text{​}}}P(X=x|X_{j}=0)). \end{array} \end{array}$$

*Replacing formulas (4) and (5) in (3) we obtain formula (2) which we use in the next example*.

**EXAMPLE 1**. *Consider the 4-node network with states X* = (*X*_1_, *X*_2_, *X*_3_, *X*_4_)*. For simplicity we assume that X is a uniform random variable that assigns equal probabilities to all x. Therefore*, *P*(*X*_*i*_ = 1) = *P*(*X*_*i*_ = 0) = 1/2 *for i* = 1, 2, 3, 4*. Define the Boolean rules as follows:*

$$\begin{array}{c} f_{1}\left(x_{2},x_{3},x_{4}\right)=x_{2}\wedge x_{3}\wedge \left(1-x_{4}\right);\\ f_{2}\left(x_{1},x_{2},x_{3}\right)=x_{1}\wedge \left(x_{2}\vee x_{3}\right);\;f_{3}\left(x_{1},x_{2}\right)=x_{1}\vee x_{2}. \end{array}$$

*Observe that the actual inputs differ from one node to the other, and that X*_4_ *can be regarded as an external input with one single output X*_1_*, and does not have a Boolean update rule f*_4_*. We can see that*

$$\begin{array}{c} \text{supp}f_{1}=\left\{\left(1,1,0\right)\right\};\text{ supp}f_{2}=\left\{\left(1,0,1\right),\left(1,1,0\right),\left(1,1,1\right)\right\};\\ \text{supp}f_{3}=\left\{\left(0,1\right),\left(1,0\right),\left(1,1\right)\right\}. \end{array}$$

*We obtain the following*.

**Table:** 

Formula (1)	*DP(i)*
*DP*(1) = *MI*(*f*_2_(*X*); *X*_1_) + *MI*(*f*_3_(*X*); *X*_1_)	*DP*(1) = 0.8601
*DP*(2) = *MI*(*f*_1_(*X*); *X*_2_) + *MI*(*f*_2_(*X*); *X*_2_) + *MI*(*f*_3_(*X*); *X*_2_)	*DP*(2) = 0.6714
*DP*(3) = *MI*(*f*_1_(*X*); *X*_3_) + *MI*(*f*_2_(*X*); *X*_3_)	*DP*(3) = 0.3601
*DP*(4) = *MI*(*f*_1_(*X*); *X*_4_)	*DP*(4) = 0.1379

*For example, to find MI*(*f*_2_(*X*);*X*_1_)*, we note that* $\underset{x\in \text{supp}f_{2}}{\sum }p_{x}=3/8$*. Since all elements of* supp*f*_2_ *have X*_1_ = 1*, it follows that*

$$\underset{x\in \text{supp}f_{2}}{\sum }P\left(X=x|X_{1}=0\right)=0$$

and

$$\begin{array}{l} \underset{x\in suppf_{2}}{\sum^{\text{​}}}P(X=x|X_{1}=1)=\underset{x\in suppf_{2}}{\sum^{\text{​}}}\frac{P(X=x,X_{1}=1)}{P(X_{1}=1)}\\ \;=\frac{P(1,0,1)}{1/2}+\frac{P(1,1,0)}{1/2}+\frac{P(1,1,1)}{1/2}\\ \;=\frac{1/8}{1/2}+\frac{1/8}{1/2}+\frac{1/8}{1/2}=\frac{3/8}{1/2}=3/4 \end{array}$$

*due to the assumption of a uniform distribution of the inputs. Then* $MI\left(f_{2}\left(X\right);X_{1}\right)=h\left(3/8\right)-\frac{1}{2}h\left(3/4\right)=0.5488$*. Similarly*, $MI\left(f_{3}\left(X\right);X_{1}\right)=h\left(3/4\right)-\frac{1}{2}\left(h\left(1\right)+h\left(1/2\right)\right)=0.3113$*. Thus*, *DP*(1) = 0.8601 *and the other DP values are obtained the same way and are included in the last column of the table above. Thus, node 1 is the most determinative in this network, followed by nodes 2, 3, and 4 in that order. This example points out that nodes with most outputs need not be the most determinative due to the Boolean function governing the node dynamics. At the same time, nodes that have the same number of outputs can lead to very different DP values*.

In the numerical results to be presented in this paper, we use the assumption of ergodicity, meaning that all input states are equally likely. Although this may not be a perfect reflection of reality, it is a most common approach in studying the dynamics of Boolean models for biological networks. For example, this assumption is used in Heckel et al. (2013), the paper that introduces the DP concept for identifying the most powerful nodes in a Boolean network. In Heckel et al. (2013) it is shown that the knowledge of the states of the most determinative nodes in the feedforward regulatory network of *E. coli* reduces the uncertainty of the overall network significantly. However, further study of non-ergodic scenarios may provide new insights.

## 3. Subnetwork size

Let us briefly describe the types of networks that will be used in simulations and for which statistical data are collected and analyzed.

The networks are obtained from Cell Collective (CC, www.cellcollective.org, Helikar et al., 2012, 2013), an interactive platform for building and simulating logical models. The database contains over 60 peer-reviewed published models of biological networks and processes. The networks are of many sizes and represent a variety of different biological processing across a number of different organisms [e.g., yeast (Irons, 2009; Todd and Helikar, 2012), flies (Marques-Pita and Rocha, 2013), humans (Conroy et al., 2014; Mendéz and Mendoza, 2016)]. Models can be simulated and analyzed directly in Cell Collective, or downloaded (as SBML or truth table files) for additional analyses in other tools. In our simulations, truth tables for a collection of networks from Cell Collective are formatted and used in a Matlab program to find the DP and subnetwork size using the above equations.

Next, we provide the actual algorithm used in conjunction with the DP of nodes to find a suitable size for the subnetwork consisting of the most determinative nodes.

**Figure 1:**
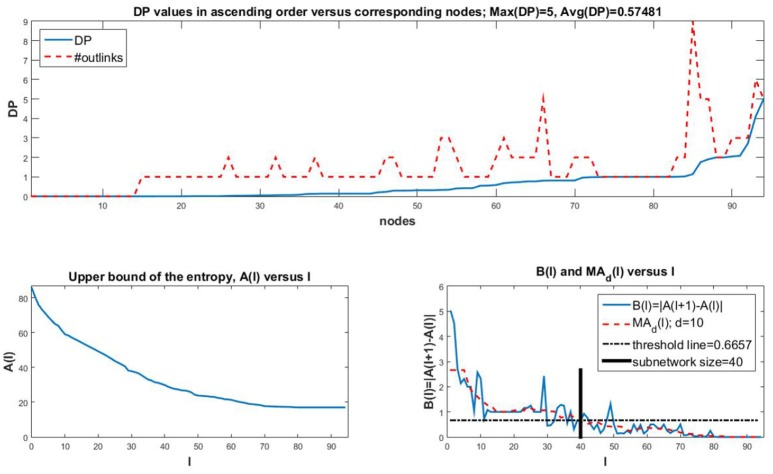
**(Top)** Sorted DP values (blue curve) for the nodes of a *T-cell Receptor Signaling* of size *n* = 94. The maximum possible values for DP, that is, the number of outputs of each node, are plotted with a red dotted curve for comparison. Notice that most nodes do not reach their maximum possible DP, and that the average DP of 0.57481 is small in comparison to the maximum DP value of 5. Thus, most nodes have a rather small DP. **(Bottom Left)** *A*(*l*) vs. *l*. Observe that the curve has an initial drop for the nodes with the largest DP values, after which the rate of convergence to a positive value is reduced as *l* increases. This indicates that once a threshold value of *l* is reached, the entropy does not decrease significantly anymore. Also, the value of *A*(*l*) for *l* = 1 is more than 4 times larger than the final value for *l* = 94. **(Bottom Right)** *B*(*l*) and *MA*_*d*_(*l*) vs. *l*. We also plot a horizontal line indicating the chosen threshold *T* for the size of the subnetwork. The bold vertical line segment indicates the subnetwork size. For this particular network, the subnetwork size is less than half of the network size.

Once each *DP*(*j*) is computed for *j* = 1, 2, …, *n*, we can sort them to identify the nodes with highest DP values. We provide an example in Figure 1 (top) where we show the DP values in ascending order for a *T-cell Receptor Signaling* network (Saez-Rodriguez et al., 2007, https://cellcollective.org/#2171/t-cell-receptor-signaling) with 94 nodes (blue curve). We also plot the maximum possible DP values (with dotted red line) given by the total number of outputs of each node, to have an understanding of how the DP compares to this maximum. Observe that if all mutual information terms would take on their maximum possible value of 1, then the DP would be the number of outputs of the node under consideration. By plotting both the DP values and the maximum possible, we can assess the “efficiency” of the node in generating the information gain in the network.

Once the DP values are sorted, we can compute the overall network entropy generated by subnetworks chosen based on top DP values of nodes. For large networks this can become a difficult task. Therefore, following the work of Heckel et al. (2013), we simplify the computations by considering an upper bound for the entropy. If we consider the collection *S*_*l*_ of the top *l* most determinative nodes, then we can compute

(6) $$\begin{array}{r} H\left(X|X_{S_{l}}\right)\leq \overset{n}{\underset{i=1}{\sum }}H\left(X_{i}|X_{S_{l}}\right),\;for\;l=1,2,3,\ldots ,n \end{array}$$

where *X*_*S*_*l*__ is the random variable whose values are the states of the nodes in *S*_*l*_. In Figure 1 (bottom left), we plot the values of the larger quantity in (6), namely $A\left(l\right)=\overset{n}{\underset{i=1}{\sum }}H\left(X_{i}|X_{S_{l}}\right)$ which is an upper bound for the entropy of the network given the top *l* nodes. Observe that for this case, subnetworks of sizes 40–50 or more (with approximation) do not yield a significant improvement of the entropy. Thus it suffices to consider less than half of the original network to be able to predict the overall network behavior with fairly low uncertainty/entropy levels. Observe also that the entropy converges to a positive value as the subnetwork size approaches the network size. This is due to the inherent uncertainty in the network based on its topology and dynamical rules.

In order to identify a precise cutoff for the subnetwork size, we follow the algorithm described next. This algorithm identifies the cutoff observed in Figure 1 (bottom right; thick vertical line segment).

*(I) Start with the sequence* {*A*(*l*), *l* = 1, 2, …, *n*}.
*(II) Construct the associated sequence of distances between consecutive terms of this sequence. That is, construct the sequence* {*B*(*l*) = |*A*(*l*+1)−*A*(*l*)|, *l* = 1, 2, …, *n*−1}.
*(III) Smooth out the sequence by applying a moving average procedure of order d, which, in our simulations it is set to* 0.1(*n*−1) *(rounded up). That is, we consider the averages over d consecutive terms of the sequence. Namely, for u* = 1, 2, …, (*n*−1)−(*d*−1), *in other words for u* = 1, 2, …, *n*−*d*, *the moving average is given by*
  (7) $$\begin{array}{r} \frac{1}{d}\overset{u+d-1}{\underset{j=l}{\sum }}B\left(j\right). \end{array}$$
  *The first and last elements of the sequence are repeated as necessary so that the final sequence of moving averages has the same length at the original sequence to be averaged. For a given *d* we label the sequence of moving averages MA_d_* = {*MA*_*d*_(*l*), *l* = 1, 2, …, *n*−1} *including all terms of formula (7) with the necessary repetitions of the first and last elements to obtain a total of n*−1 *terms. An even d value generates an odd number of repeated elements, which leads to one extra repetition of the last element as opposed to the repetitions of the first element (see MA*_4_ *in the example below)*.
  *For instance, if the input sequence of B(l) values is* {10, 9, 8, 7, 6, 5, 4, 3, 2, 1} *then some sample {MA*_*d*_} *sequences are*
  $$\begin{array}{lll} MA_{3} & = & \left\{9,9,8,7,6,5,4,3,2,2\right\}\\ MA_{4} & = & \left\{8.5,8.5,7.5,6.5,5.5,4.5,3.5,2.5,2.5,2.5\right\}\\ MA_{5} & = & \left\{8,8,8,7,6,5,4,3,3,3\right\}. \end{array}$$
  *For example, to clarify even further, in the case of MA*_3_ *and u* = 1, *formula (3) generates* 1/3(*B*(1)+*B*(2)+*B*(3)) = 9. *However, since n*−*d* = 10−2 = 8 *we repeat the first and last terms of the sequence given by (3), so that MA*_3_(1) = *MA*_3_(2) = 9. *Similarly, MA*_3_(9) = *MA*_3_(10) = 1/3(*B*(8)+*B*(9)+*B*(10)) = 2.
*(IV) Set T, the threshold for finding the size of the subnetwork. In simulations we use* $T=\frac{1}{4}\max\left(MA_{d}\right)$. *More precisely, starting with l* = 1, *we increase l by one unit until we reach a value L for which the following conditions are satisfied*
  (8) $$\begin{array}{r} MA_{d}\left(L\right)\leq T\;and\;\frac{1}{d}\overset{\min\left(L+d-1,n-1\right)}{\underset{j=L}{\sum }}MA_{d}\left(j\right)\leq T. \end{array}$$
  *That is, the values of the MA_d_ sequence drop below the threshold T and the average variance of the next d values of MA_d_ is also less than the threshold T*.
*(V) The subnetwork consists of the nodes with the L highest DP values*.

The results are dependent on how one sets the parameters *d* and *T*. The larger the *d* value, the smoother the *MA*_*d*_ sequence, and thus the conditions (8) tend to be satisfied for smaller values of *l*. The same happens if *T* is sufficiently large. On the other hand, larger moving average order *d* means losing some of the intrinsic variation of data. Therefore, we need to be aware of the tradeoff between accuracy and details of the data, as is customary in network modeling and simulation.

In Figure 1 (bottom right), this algorithm with *d* = 0.1(*n*−1) and $T=\frac{1}{4}\max\left(MA_{d}\right)$ generates a minimal subnetwork size of 40 nodes with the largest DP. This is less than half of the network size. We notice that the threshold *T* is approximately $\frac{1}{4}\max\left(MA_{d}\right)=\frac{1}{4}\cdot 2.8=0.7$.

**Figure 2:**
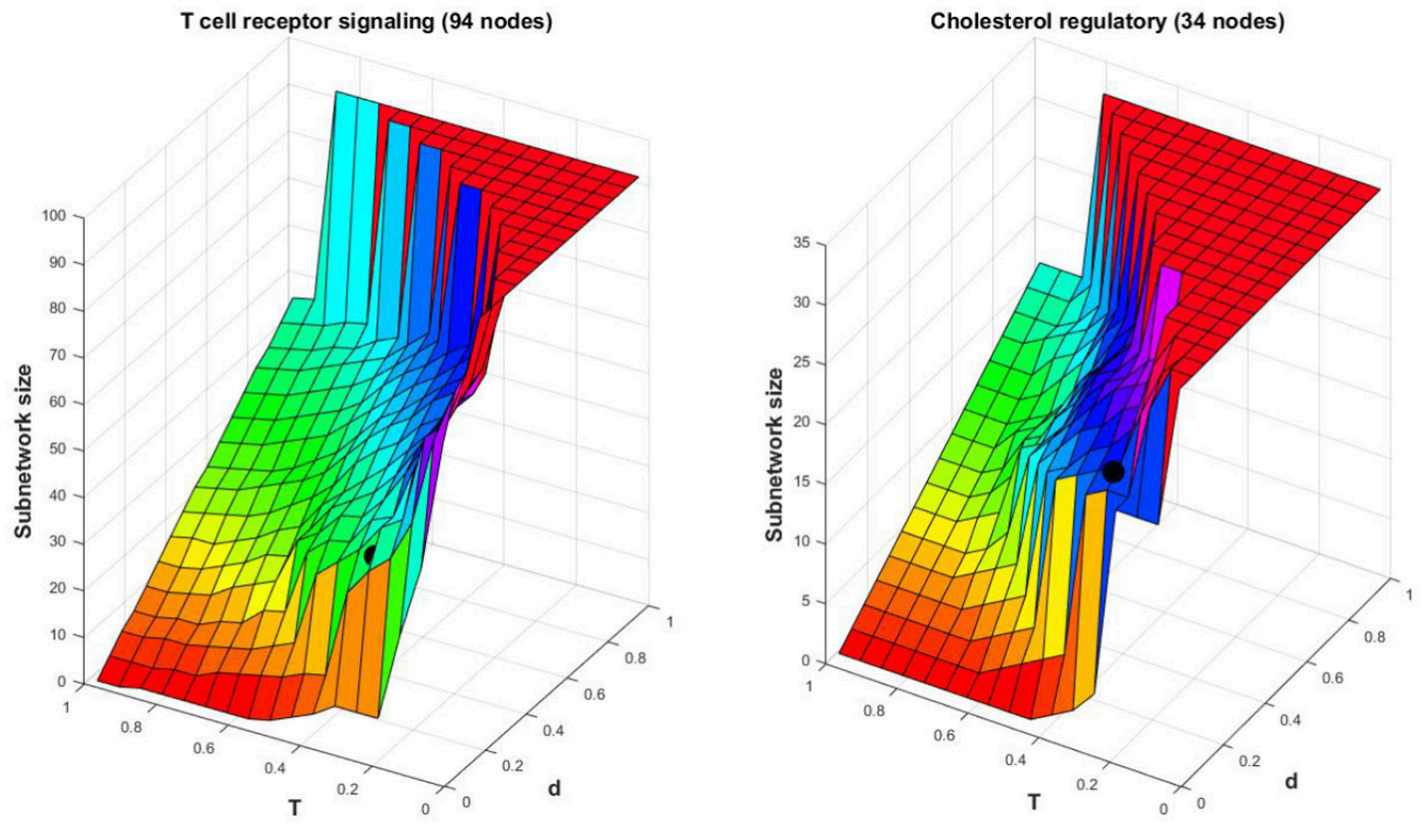
Surface plot for *L* vs. a grid of values of *d* and *T*. The black dot represents the point $L,d=0.1\left(n-1\right),T=\frac{1}{4}\max\left(MA_{d}\right)$ for the *T-cell Receptor Signaling* model with *L* = 40, and for the *Cholesterol Regulatory Pathway* model with *L* = 22.

To see how the two parameters *d* and *T* affect the size *L* of the subnetwork, we compute *L* for a grid of values of *d* and *T* for two networks that will be used as examples in the next section too. Two sample surfaces are shown in Figure 2. The black dot indicates the actual *L* value obtained with this procedure for *d* = 0.1(*n*−1) and $T=\frac{1}{4}\max\left(MA_{d}\right)$ considered in the simulations. As expected, the values of *L* increase with an increase of the two parameters, and the surfaces are similar in shape with mild variations.The choice of *d* = 0.1(*n*−1) used in simulations generates subnetworks that do not surpass 60% of the network size with approximation. We will see that this is sufficient to identify a good fraction of biologically important nodes in several networks from the Cell Collective (Helikar et al., 2012, 2013).

We explore other networks in the next section, however, we will provide graphs related to the networks considered so far and add one more network of small size.

## 4. Numerical results and analysis

### 4.1. Simulations and statistical analysis

We apply the procedure explained in the previous section to a number of networks available in Cell Collective (Helikar et al., 2012, 2013). We summarize the results below and supplement with suitably chosen graphs. For each network shown in graphs, we plot the sorted DP values for all nodes, the upper bound for the entropy, *A*(*l*) vs. *l*, and the elements of the algorithm for finding the subnetwork size, namely *B*(*l*) and *MA*_*d*_(*l*) vs. *l* with a horizontal line at the threshold value *T* that indicates the subnetwork size.

The graphs of *A*(*l*) consist of a curve that decreases to zero or a value that stabilizes for large values of *l* in most cases. A typical example is the one considered in the previous section for the *T-cell Receptor Signaling* network in Figure 1. This behavior is very similar to the results obtained in Heckel et al. (2013), the paper that inspired this work, for a feedforward regulatory network in *E. coli*. Notice that *A*(*l*) stabilizes at a positive value for large *l* and does not converge to zero. In general, since *A*(*l*) is an upper bound for the entropy as seen in inequality (6), it may not approach zero. On the other hand, the entropy itself is the expected value of a random variable as indicated in Definition 2, and therefore it may be non-zero.

**Figure 3:**
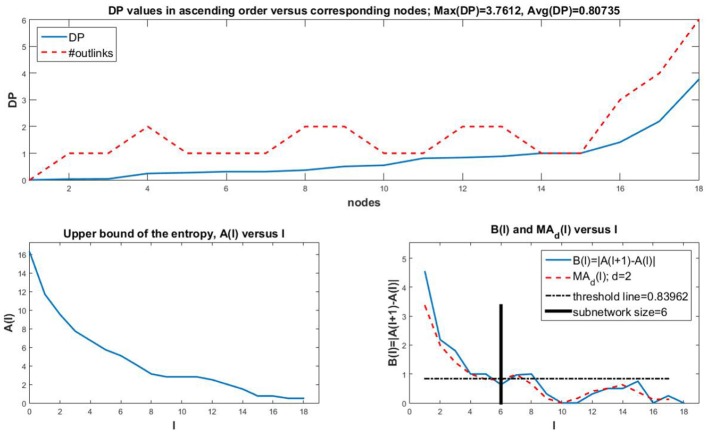
Analog of Figure 1 for the *Oxidative Stress Pathway* network with *n* = 18. The average DP is larger than for the *T-cell Receptor Signaling* network, which can be expected in a smaller network where nodes may incorporate more information to be used in the network. The maximum DP is smaller though. Observe that here, *A*(*l*) decreases to a value close to zero along a non-linear curve. The subnetwork size is a third of the network size, so it is smaller as a fraction of the network in comparison to the *T-cell Receptor Signaling* network where the subnetwork is about 42% of the network size.

**Figure 4:**
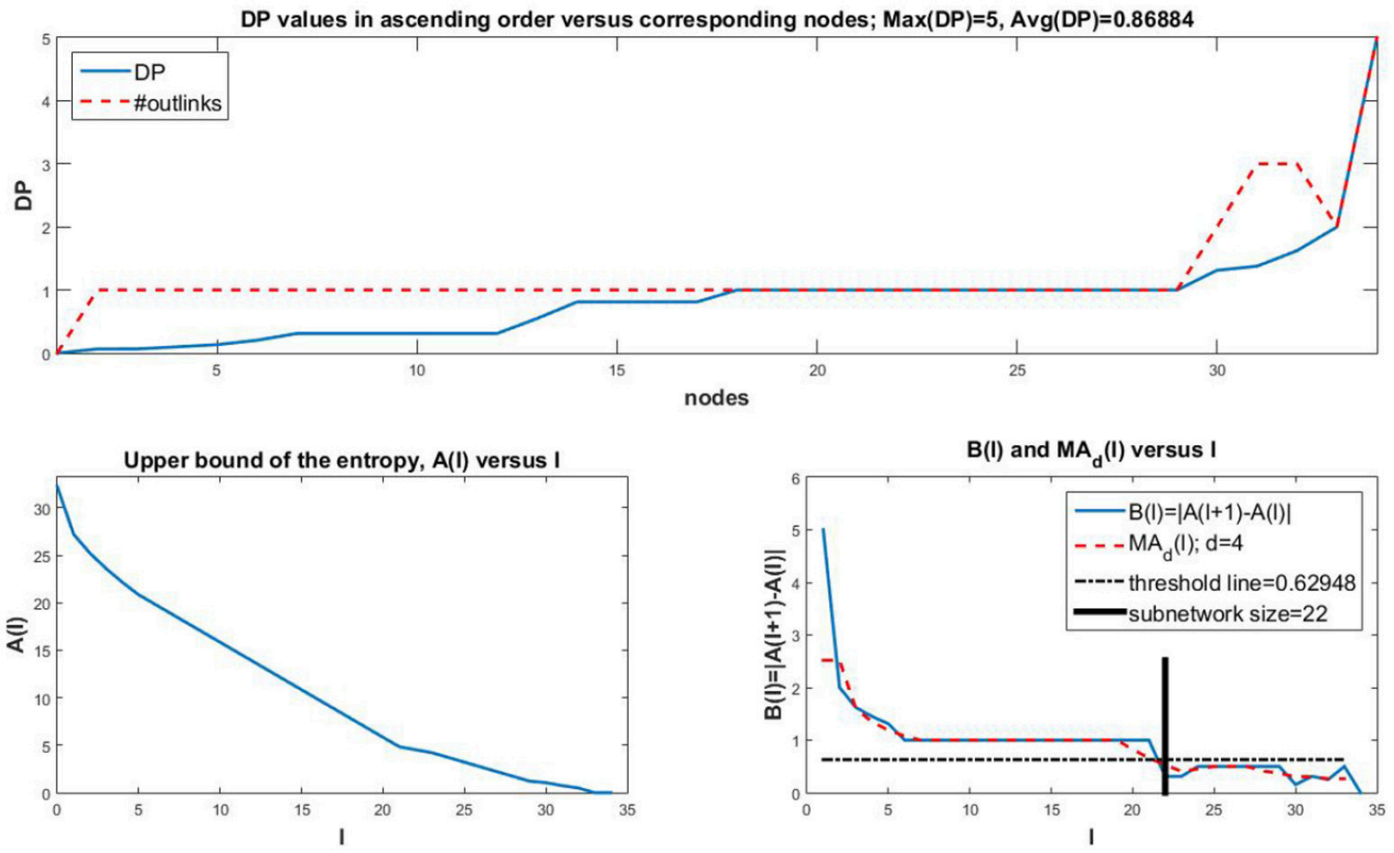
The *Cholesterol Regulatory Pathway* network consists of *n* = 34 nodes. The maximum and average DP are comparable to the *Oxidative Stress Pathway* network. The upper bound *A*(*l*) for the *Cholesterol Regulatory Pathway* network has an almost linear decrease to zero. Therefore, the subnetwork size of 22 is larger than in previous cases in comparison to the network size, representing about 65% of the network.

A couple of variations are shown as well. In Figure 3 we consider a small *Oxidative Stress Pathway* network with 18 nodes (Sridharan et al., 2012, https://cellcollective.org/#3512/oxidative-stress-pathway). The subnetwork size is a third of the network size. In Figure 4 we show similar graphs for a medium sized *Cholesterol Regulatory Pathway* network with 34 nodes (Kervizic and Corcos, 2008, https://cellcollective.org/#2172/cholesterol-regulatory-pathway). In this case, the upper bound *A*(*l*) approaches zero rather slowly at an almost linear rate, therefore the subnetwork size is larger when compared to the whole network, namely about 65% of the entire network.

Next, we summarize the data obtained from a total of 36 networks and generate some statistical information. Four networks are significantly larger than the others: signal transduction in fibroblast cells with 130 nodes, interleukin-1 signaling with 103 nodes, signal transduction in a macrophage with 302 nodes, and T-cell receptor signaling with 94 nodes. We consider them “outliers” and explore some statistics on the remaining 32 networks to avoid skewed results. We hope to be able to expand the list of large networks in the future and include them in the analysis.

**Figure 5:**
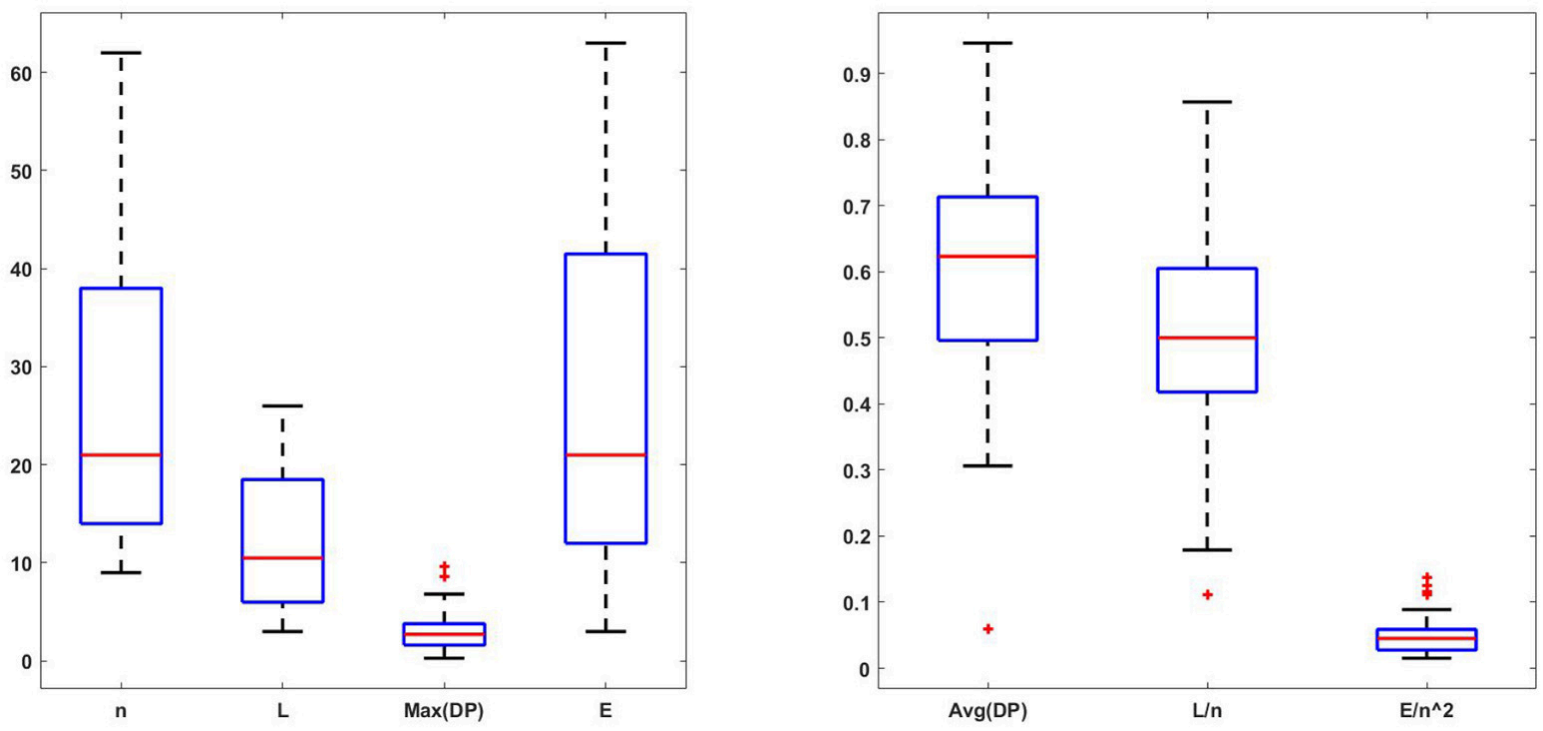
Boxplots for the network size *n*, subnetwork size *L*, maximum DP, average DP, ratio *L*/*n*, number of edges *E*, and the ratio *E*/*n*^2^. They are grouped based on similar magnitudes. The boxplots for *n* and *E* are very similar. It appears that most subnetwork sizes are fairly small even for larger *n* or *E* values, so the subnetwork sizes may not increase with the network size or the number of edges. The maximum DP seems to be fairly large. The average DP is rather small for all networks regardless of their sizes. The boxplot for *L*/*n* indicates that most subnetworks represent less than 60% of the original network. The number of edges *E* is small in comparison to the total number of possible edges in the network due to small values of the quantity *E*/*n*^2^∈[0, 0.13]. This suggests that these networks do not have too many links.

We provide boxplots for seven numerical characteristics obtained from the network data: network size *n*, subnetwork size *L*, maximum DP values, average DP values, ratio *L*/*n*, number of links or edges in the network, *E*, given by the total number of inputs or outputs for all nodes, and *E*/*n*^2^ as the ratio between the edges and total number of possible edges, taking into account that self-inputs are allowed. The results are shown in Figure 5. We choose to separate them due to the different ranges of values. Observe that most subnetwork sizes are fairly small even for larger networks or more edges, so the subnetwork sizes may not increase with the network size or the number of edges. The number of nodes and the number of edges have similar boxplots. The maximum DP can be fairly large; however it is not clear yet if this fact is related to the network size, or the number of edges. We will explore the idea in what follows. Finally, the average DP is rather small for all networks, regardless of their sizes. Also, most of the ratios *L*/*n* of the subnetwork size vs. the network size are less than 60%.

**Figure 6:**
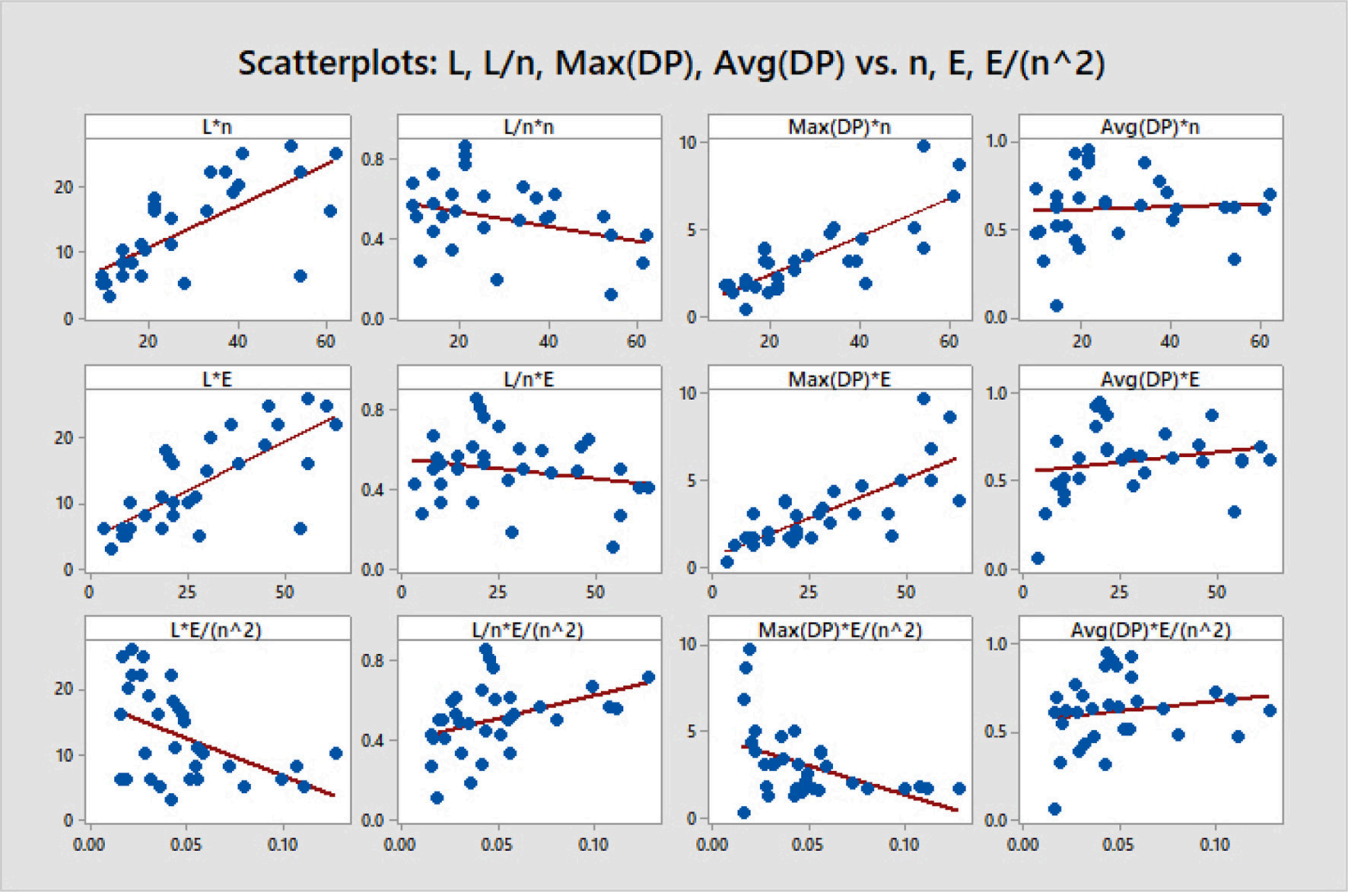
Scatter plots and fitted lines for the identification of possible correlations between *L, L*/*n*, Max(DP), Avg(DP), and the parameters *n, E, E*/*n*^2^. The equations for the fitted lines are listed in Table 1. There are no observable strong correlations and this is confirmed by the coefficients of determination in Table 1. Weak correlations are noticed for the increasing subnetwork size *L* as a function of *n* or *E*, and the increasing Max(DP) as a function of the same two parameters *n* and *E*. We also notice the decreasing trend of the ratio *L*/*n* with increased network size *n* or number of edges *E*, which suppports our observations from the boxplots.

We also explore the dependencies between the numerical characteristics considered in Figure 5, by generating a number of scatter plots with corresponding fitted regression lines. In particular, we want to see if there are correlations between *L, L*/*n* or the maximum DP and average DP vs. the network parameters *n, E, E*/*n*^2^. We find that there is no evidence of strong correlations between the variables, except for *L* vs. *n, E* and maximum DP vs. *n, E*. The scatter plots are shown in Figure 6 and the corresponding fitted lines and coefficients of determination *R*^2^ are listed in Table 1. Note that there is no strong linear (or non-linear) relationship; however we note the increasing trend in both subnetwork size L and maximum DP with increased *n* and *E*. On the other hand we see that the average DP does not depend on the parameters and that the ratio *L*/*n* decreases with increased *n, E*, which supports the observations from the boxplots.

**Table 1:** Fitted lines and coefficients of determination *R*^2^ corresponding to the scatter plots of Figure 6.

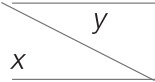	**L**	***L*/*n***	**Max(DP)**	**Avg(DP)**
*n*	*y* = 4.167+0.3187*x*	*y* = 0.6057−0.0037*x*	*y* = 0.176+0.1088*x*	*y* = 0.5906+0.0009*x*
	*R*^2^ = 51.1%	*R*^2^ = 11.9%	*R*^2^ = 66.2%	*R*^2^ = 0.5%
*E*	*y* = 4.638+0.2985*x*	*y* = 0.5622−0.002*x*	*y* = 0.6277+0.0912*x*	*y* = 0.5493+0.0024*x*
	*R*^2^ = 56.9%	*R*^2^ = 4.7%	*R*^2^ = 59.1%	*R*^2^ = 4.8%
*E*/*n*^2^	*y* = 18.24−115*x*	*y* = 0.3961+2.315*x*	*y* = 4.697−33.29*x*	*y* = 0.5616+1.118*x*
	*R*^2^ = 23%	*R*^2^ = 16.1%	*R*^2^ = 21.4%	*R*^2^ = 2.8%

*The coefficients are generally small, the maximum values being observed for L vs. n, E and for Max(DP) vs. n, E. However, the maximum coefficient is only 66.2%, which suggests weak correlations at best*.

Thus, the given data do not suggest a specific strong relationship between the numerical characteristics; however they allow us to observe trends and support some of the previous observations in the boxplots. Our samples are quite small, so it would be useful to continue adding new networks to the collection considered in this paper, to overcome the possible inaccuracies due to small sample size. The change of parameters in the network size algorithm leads to a fairly similar change in the subnetwork size for different networks as seen in Figure 2, suggesting a correlation between the choice of parameters and the subnetwork size *L*. We expect that other possible variables or attributes that are intrinsic to the actual topology or dynamics of networks may have a stronger correlation with the DP values. Some of these attributes are connectivity (in-degree), number of outputs (out-degree), path length and other topological measures, canalizing depth, ratio of canalizing functions, or average bias of outputs (Albert and Barabasi, 2002; Kochi et al., 2014; Wohlgemuth and Matache, 2014). We plan on exploring them in great detail in future research to shed more light on possible relationships with the variables in Figures 5, 6.

The observed general low DP values is what we expect in an equilibrium situation. It has been shown that the correlations between nodes become high only when facing a transition (Gorban et al., 2010; Censi and Calcagnini, 2011; Mojtahedi et al., 2016). It is possible that the simple node level hierarchy coming from mutual information might benefit from a study of at least some complex graph analysis descriptors such as in-degree, out-degree, betweenness and closeness centrality of the nodes that keep track of the role played by the nodes in the system they are embedded into (Csermely et al., 2005; Kovacs et al., 2010). In the next section we complement our analysis with a brief graph-theoretical perspective that is relevant in signaling networks (Di Paola and Giuliani, 2015).

### 4.2. Determinative power and topological attributes

We will focus on some topological attributes or measures associated with the nodes of a BN that may provide more information on the magnitude of the DP values. Given a BN, [*n*]: = {1, 2, …, *n*}, and an arbitrary node *j*∈[*n*], we consider the connectivity or the number *k*_*j*_ of inputs of the node *j* (the in-degree), the number *o*_*j*_ of outputs of the node *j* (the out-degree), together with several measures of centrality of node *j* as defined below.

**DEFINITION 3**. *A sequence of distinct nodes P*(*i*_1_, *i*_*m*_) = {*i*_1_, *i*_2_, …*i*_*m*_} *of a BN with the property that i*_*k*_ *is an input to i*_*k*+1_ *for any k* = 1, 2, …, *m*−1*, is called a path of length m*−1 *from the source node i*_1_ *to the destination node i*_*m*_. *Thus, the distance between the two nodes along this path is d*(*i*_1_, *i*_*m*_) = *m*−1.

There could be multiple paths between two nodes, possibly with the same length. We are interested in the shortest path length between nodes. Observe that the shortest path length may differ if we switch the source and the destination nodes, so we may have *d*(*i*_1_, *i*_*m*_)≠*d*(*i*_*m*_, *i*_1_). On the other hand, if there is no path from node *i* to node *j* then *d*(*i, j*) = 0.

For a given node *i*∈[*n*], let us consider the following quantities. We use the notation |*A*| to denote the cardinality of the set *A*, in other words the number of elements in that set. Let

$$\begin{array}{l} A_{in}(i)=|\{j\in [n]:j\neq iandthereexistsapathP(j,i)\}|,\\ \;F_{in}(i)=\underset{j\neq i}{\sum^{\text{​}}}d(j,i),\\ A_{out}(i)=|\{j\in [n]:j\neq iandthereexistsapathP(i,j)\}|,\\ \;F_{out}(i)=\underset{j\neq i}{\sum^{\text{​}}}d(i,j). \end{array}$$

If *A*_*in*_(*i*) = 0 then *F*_*in*_(*i*) = 0, and similarly, if *A*_*out*_(*i*) = 0 then *F*_*out*_(*i*) = 0. The quantities *F*_*in*_(*i*), *F*_*out*_(*i*) could be regarded as measures of the farness of node *i* from the other nodes in the network. The reciprocal of farness is a measure of closeness. If we multiply the closeness by the fraction of the sources or destinations of node *i* we obtain the following definitions of closeness centrality.

**DEFINITION 4**. *The in-closeness centrality of node i*∈[*n*] *is the quantity*

$$C_{in}\left(i\right)={\left(\frac{A_{in}\left(i\right)}{N-1}\right)}^{2}\frac{1}{F_{in}\left(i\right)},\;if\;A_{in}\left(i\right)\neq 0,$$

*and C*_*in*_(*i*) = 0 *otherwise*.

*Similarly, the out-closeness centrality of node i*∈[*n*] *is the quantity*

$$C_{out}\left(i\right)={\left(\frac{A_{out}\left(i\right)}{N-1}\right)}^{2}\frac{1}{F_{out}\left(i\right)},\;if\;A_{out}\left(i\right)\neq 0,$$

*and C*_*out*_(*i*) = 0 *otherwise*.

A second measure of centrality is the betweenness centrality, which measures how often each node appears on a shortest path between two nodes in the network. Given three distinct nodes *i, j, k*, let *N*_*jk*_ be the total number of shortest paths from *j* to *k*, and *N*_*jk*_(*i*) the number of those paths that pass through node *i*.

**DEFINITION 5**. *The betweenness centrality of node i*∈[*n*] *is the quantity*

$$BC\left(i\right)=\underset{j,k\neq i}{\sum }\frac{N_{jk}\left(i\right)}{N_{jk}}.$$

*The summation is over all nodes j, k for which N*_*jk*_≠0*, meaning there exists at least a path between them*.

We compute the topological attributes of nodes for the individual networks considered in previous figures, namely the *T-cell Receptor Signaling*, the *Oxidative Stress Pathway*, and the *Cholesterol Regulatory Pathway*. However, we are also adding one of the outlier networks, namely the signal transduction in fibroblast cells network with 130 nodes. The *Fibroblast Signaling* network has been investigated before in various publications (Kochi and Matache, 2012; Kochi et al., 2014; Matache and Matache, 2016; Puniya et al., 2016).

**Figure 7:**
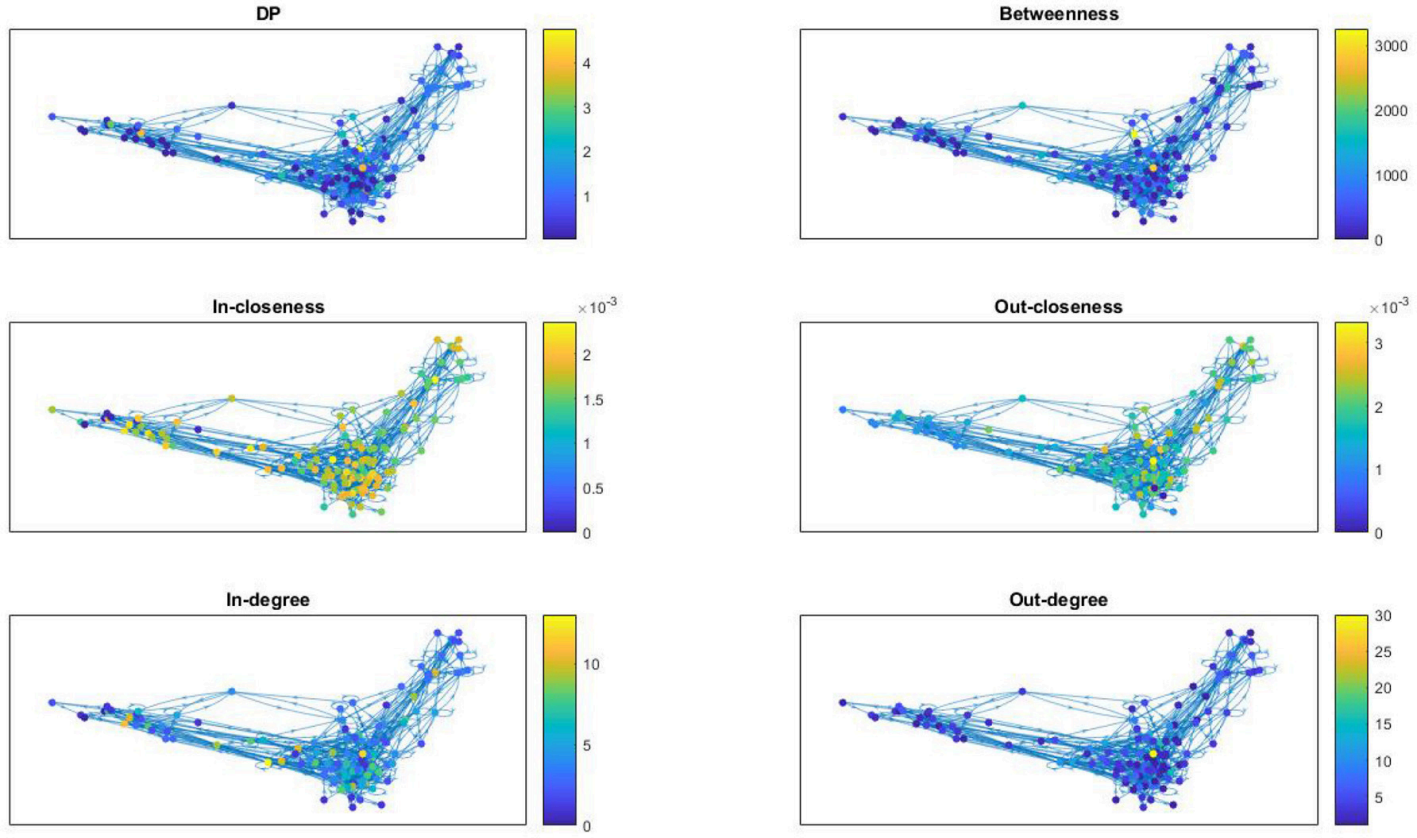
*Fibroblast Signaling* network: visualization of node attributes.

**Figure 8:**
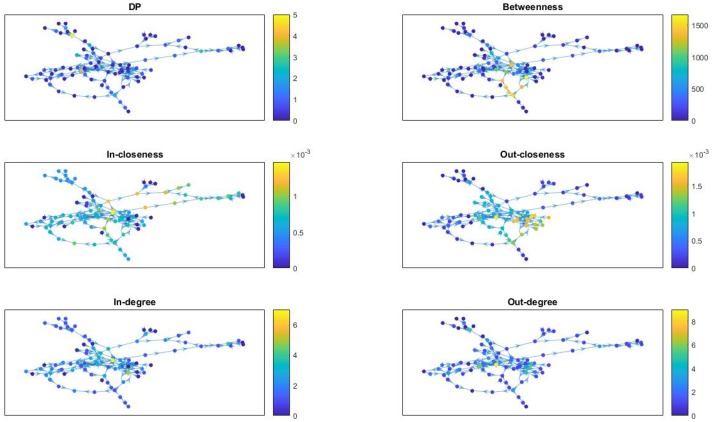
*T-cell Receptor Signaling* network: visualization of node attributes.

**Figure 9:**
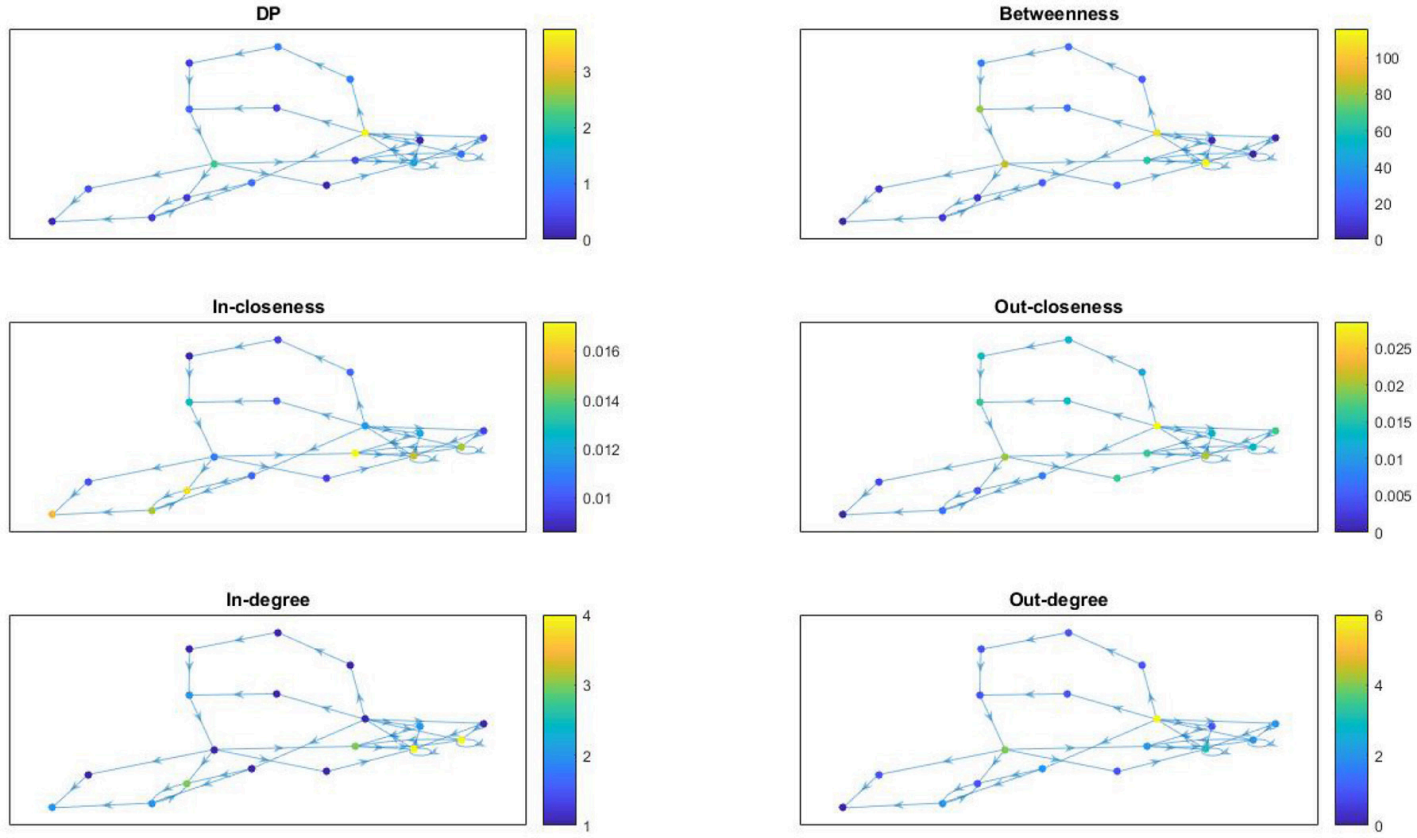
*Oxidative Stress Pathway* network: visualization of node attributes.

**Figure 10:**
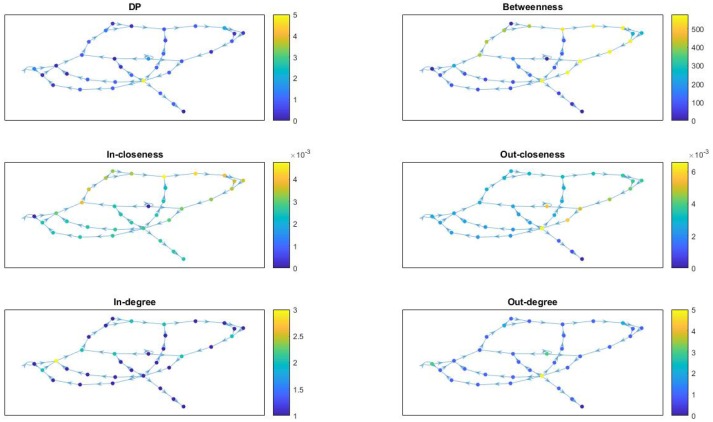
*Cholesterol Regulatory Pathway* network: visualization of node attributes.

In Figure 7 we provide network visualizations for each of the node attributes described in this section for the *Fibroblast Signaling* network. They are presented in the following order: DP, betweenness centrality, in-closeness centrality, out-closeness centrality, in-degree, and out-degree. The node color is proportional to the magnitude of these measures: dark colors for low values and light colors for large values. This type of visualization offers an overall view of the network's most central nodes, as well the nodes with most connections, or the nodes with highest DP values, thus identifying, to some extent, the role played by the nodes in the network they are embedded into. Similar graphs are shown in Figure 8 for the *T-cell Receptor Signaling* network, in Figure 9 for the *Oxidative Stress Pathway* network, and in Figure 10 for the *Cholesterol Regulatory Pathway*.

**Figure 11:**
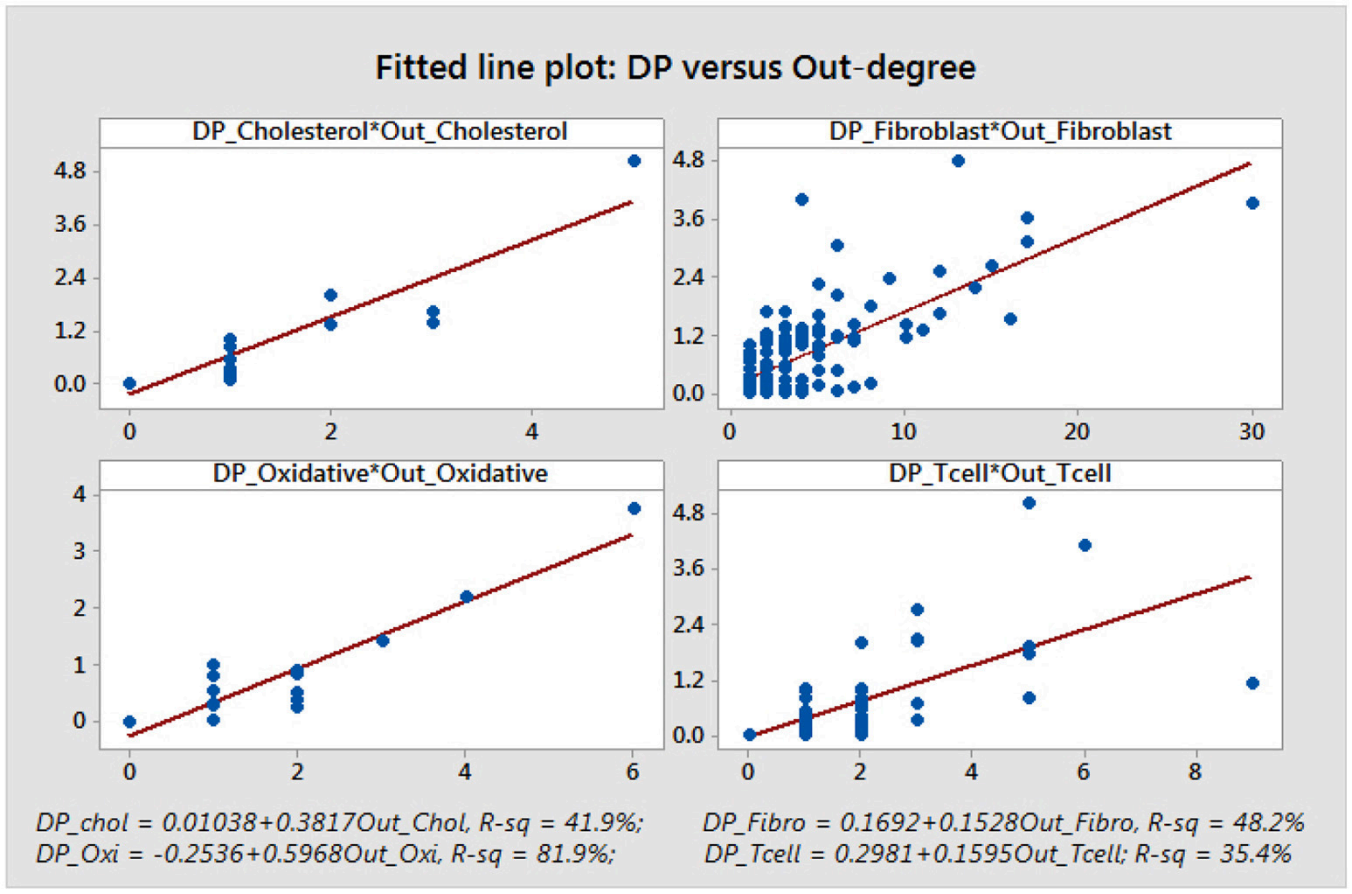
The scatter plots suggest some correlation between the DP values and the number of outputs. However, we can observe that there might be situations where a large DP does not correspond to a large number of outputs. There can also be situations where the DP is small even though the node has more outputs.

**Figure 12:**
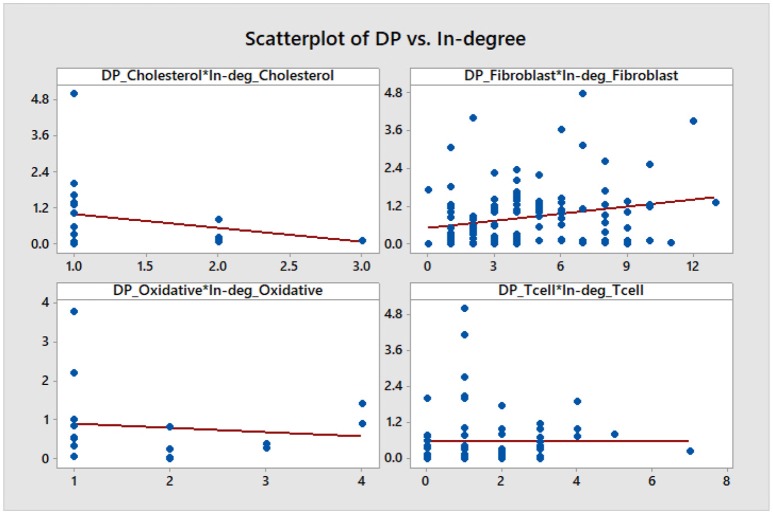
Simple scatter plots for DP vs. in-degree. *R*^2^∈[0%, 7.3%].

**Figure 13:**
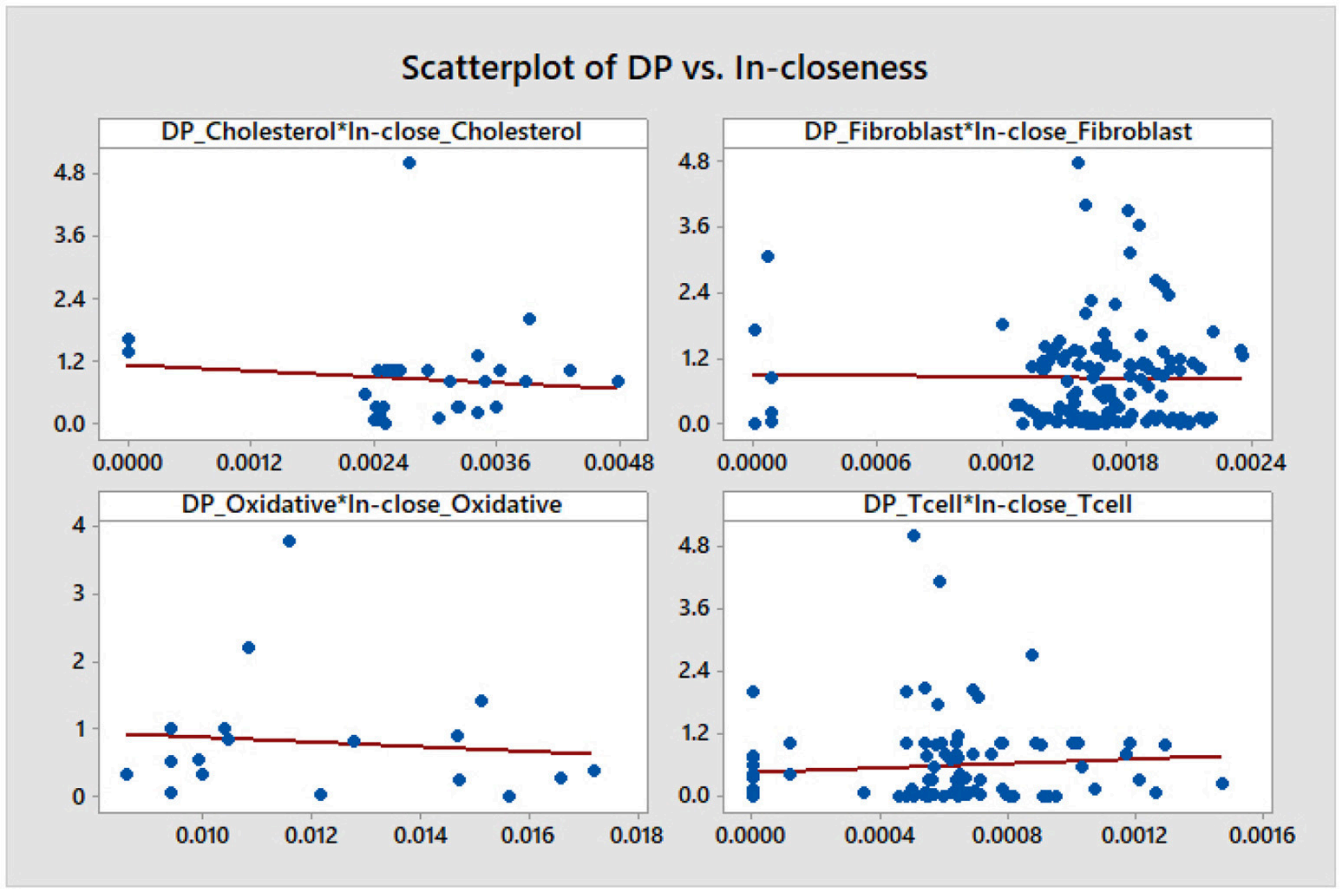
Simple scatter plots for DP vs. in-closeness centrality. *R*^2^∈[0%, 1.3%].

**Figure 14:**
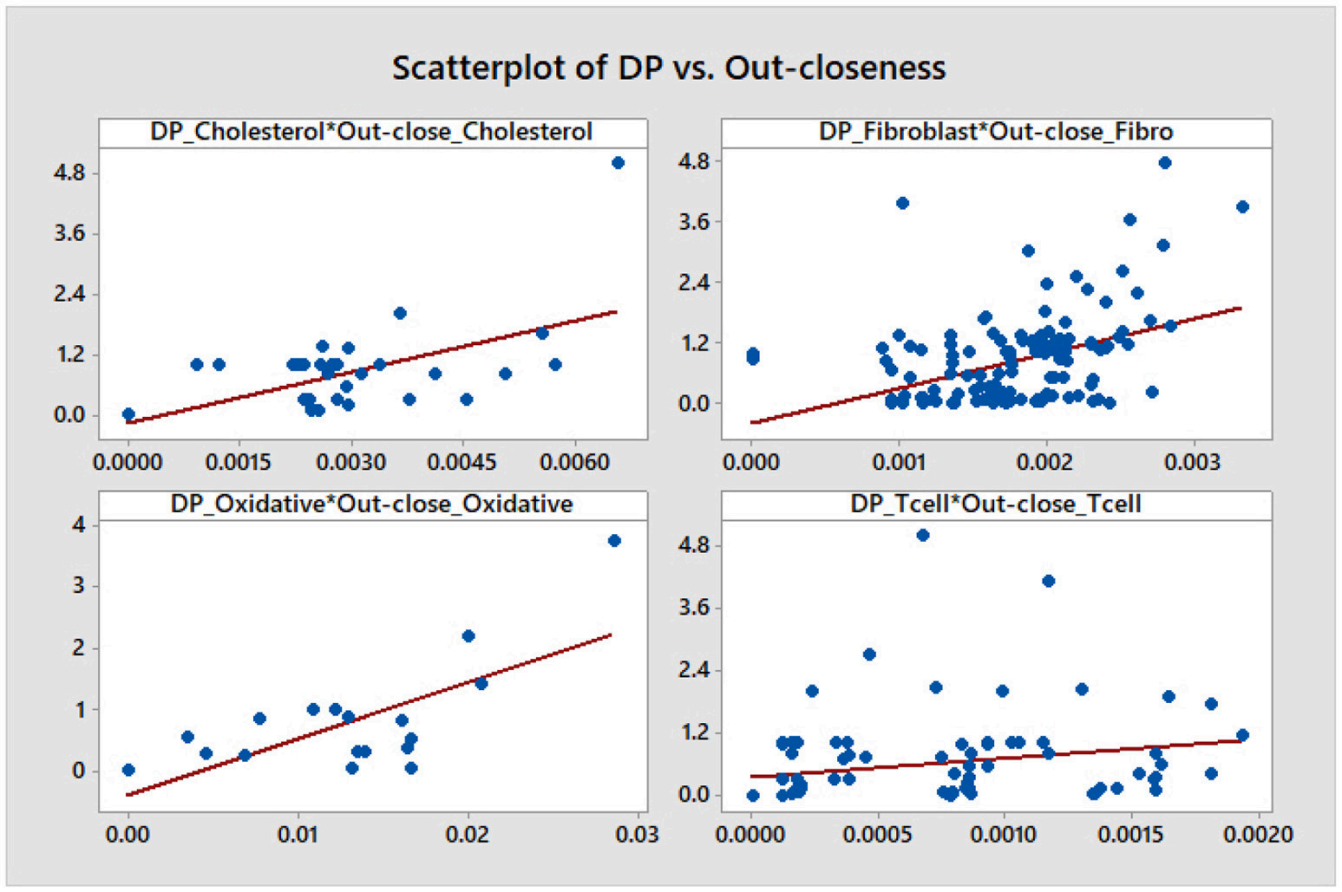
Simple scatter plots for DP vs. out-closeness centrality. *R*^2^∈[5.7%, 48%], where 48% corresponds to the *Oxidative Stress Pathway* network.

**Figure 15:**
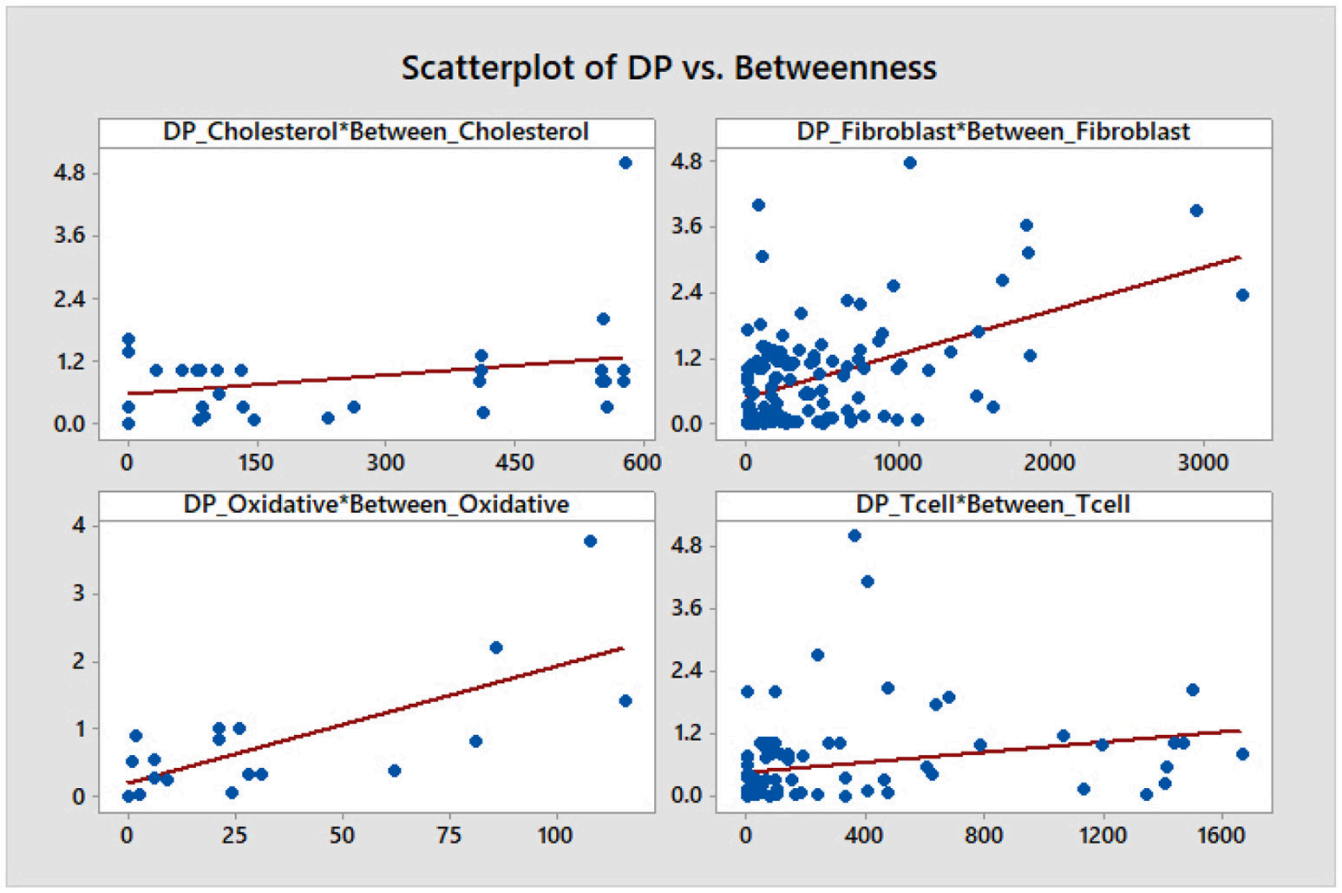
Simple scatter plots for the DP vs. betweenness centrality. *R*^2^∈[6.5%, 52%], where 52% corresponds to the *Oxidative Stress Pathway* network.

We note that, aside from some similarities between the DP and the out-degree graphs which are expected given the definition of the DP as a summation of mutual information terms over all outputs of a given node, there is no other significant correlation. This is confirmed by a statistical analysis of the topological data. We include scatter plots with corresponding fitted regression lines for the DP as a function of the out-degree in Figure 11, together with the corresponding coefficients of determination *R*^2^. The plots indicate that there might be nodes with high DP and fewer outputs, and also nodes with low DP and a larger number of outputs. In section 4.3 we relate this fact to the biological relevance of the nodes with large DP values. We provide simple scatter plots for DP as a function of the other topological measures indicating only the ranges of values of *R*^2^ in Figures 12–15. The coefficient of variation is quite small in most cases. The largest values correspond to the DP vs. out-closeness and betweenness centrality of the smallest network, the *Oxidative Stress Pathway* network. However, even these values are around 50%. We also conclude that for the four networks under consideration the DP is not correlated with any of the other topological measures.

Thus, further analyses need to be pursued, including other topological aspects in conjunction with various dynamical measures. For example, it has been shown that the location of nodes in the network may be crucial for identifying enzymes whose elimination may have lethal effects in certain metabolic networks (Palumbo et al., 2005, 2007). In that case the metabolites are considered the nodes of the network, whereas the enzymes are the links between nodes. Therefore, it may be of further interest to explore other node location measures.

### 4.3. Biological relevance of the most determinative nodes

Aside from providing a method for finding a subnetwork with a fairly low impact on the overall entropy of the system, the DP method identifies biologically significant nodes among the top DP values. To support this statement we analyze biological relevance of the top DP nodes.

We focus on the particular networks shown in the figures so far, namely *Fibroblast Signaling, T-cell Receptor Signaling, Oxidative Stress Pathway, Cholesterol Regulatory Pathway*. These are all intercellular networks found in many different organisms. We are interested in the biological relationship between high DP and the nodes' biological importance in the cell. In our analysis, we provide most information on the larger networks from among these four, namely *Fibroblast Signaling* and *T-cell Receptor Signaling*, and a shorter summary for the other two networks.

We start with a few notes on *Fibroblast Signaling*. To investigate in more detail whether nodes with high DP values are influential, we compare these nodes with the 32 most influential nodes identified under different environmental conditions in a previously published study by Puniya et al. (2016). We compare these most influential nodes with the top 10, 20, 30, 40, 50, and 60 nodes having high DP values in our analysis. We obtain an overlap of 70%, 65%, 50%, 47%, 38%, and 33%, respectively. Among the top 20 nodes having high DP values, 13 were previously identified as the most influential. Among the top 10, we find only one node which was previously identified as less influential. Similarly, in the top 20, 30, 40, 50, and 60 nodes, the distribution of the previously identified less influential nodes are 2, 3, 4, 8, and 13 respectively. This comparison suggests that the majority of nodes having high DP values (>65% in the top 20) are also identified as most influential when perturbed under different environmental conditions by Puniya et al. (2016). Therefore, these nodes may be involved in crucial biological functions.

Furthermore, we perform functional analyses of these nodes having high DP values. We provide information on all four networks under consideration.

**Figure 16:**
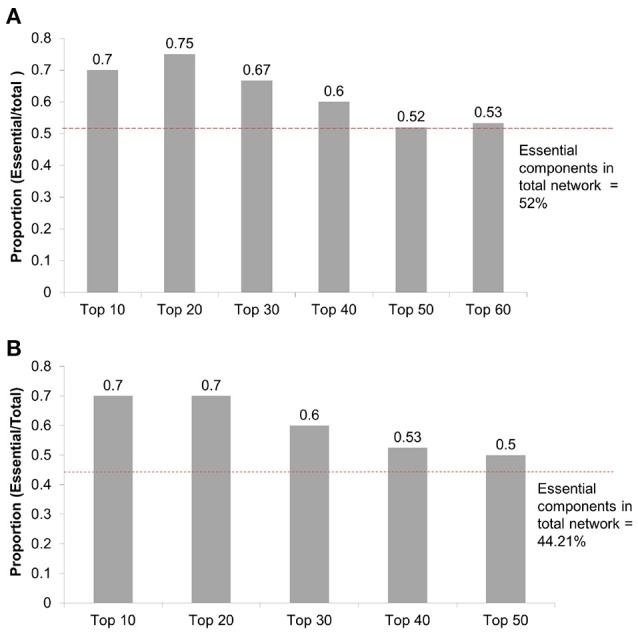
**(A)** Distribution of essential nodes among top nodes having high DP values in the *Fibroblast Signaling* network with *n* = 130 nodes. Y-axis: Number of essential nodes among the selected nodes divided by number of selected nodes (top DP nodes). The red horizontal line indicates the proportion of essential nodes found in the whole network. **(B)** Distribution of essential nodes among top nodes having high DP values in the *T-cell Receptor Signaling* network with *n* = 94 nodes.

**Figure 17:**
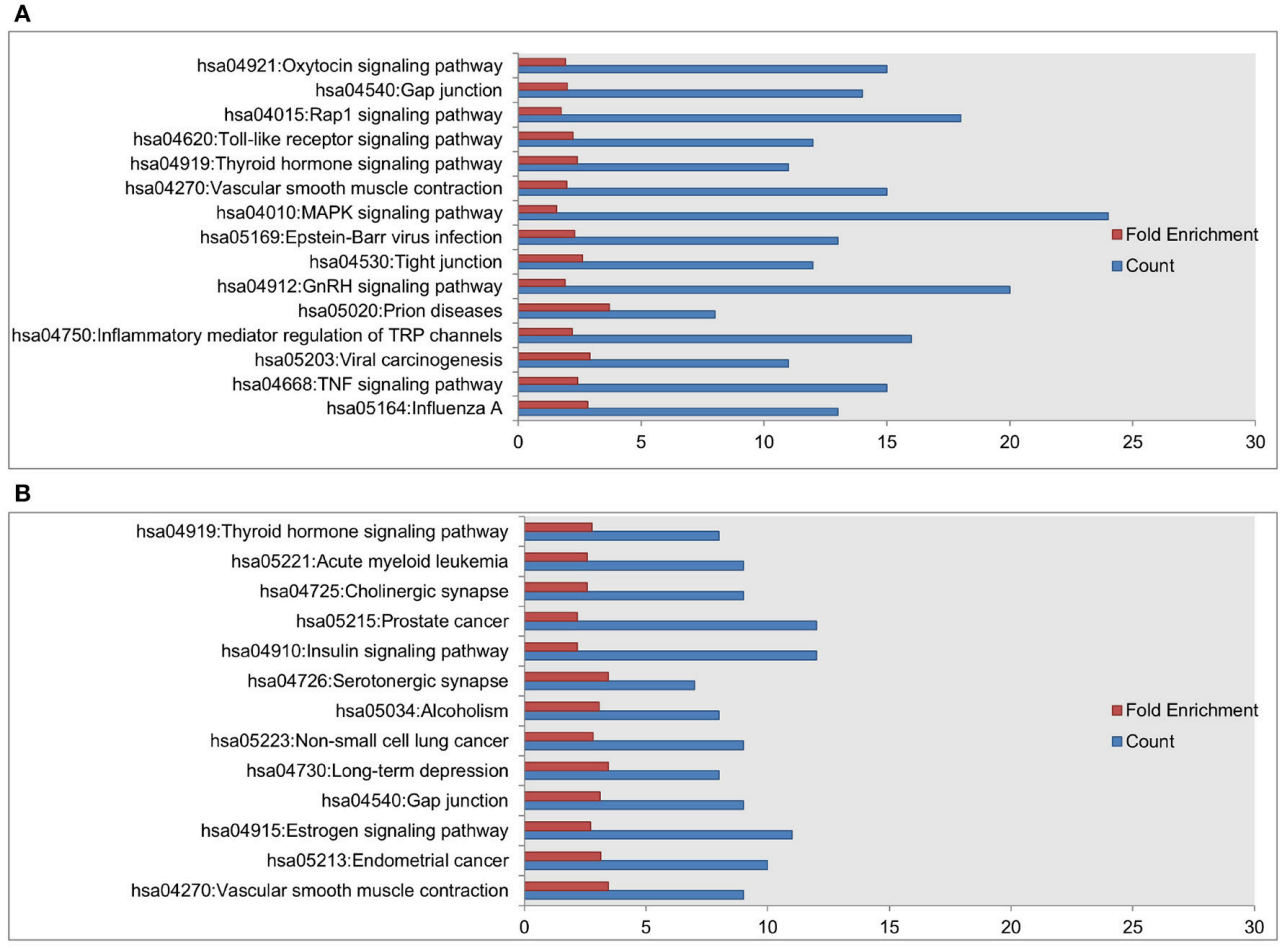
Enriched KEGG pathways among the top 20 selected nodes having high DP values in the **(A)** *Fibroblast Signaling* network with 130 nodes and **(B)** *T-cell Receptor Signaling* network with 94 nodes. The values on the x-axis correspond to fold enrichment and the total number of genes found in the KEGG pathway. The enriched pathways are given on the y-axis.

1. **Methods**
  Gene essentiality data are obtained from the Online GEne Essentiality (OGEE) database version 1 that was downloaded on July 20, 2015 (Chen et al., 2012, 2017). Essential genes are deemed to be critical for cellular function and survival. As such, if an essential gene is removed (or knocked-out), it results in inviability. The OGEE database lists 7,168 genes as essential and 6,985 genes as conditionally (under specific environmental conditions) essential for humans, and was compiled using 18 different datasets of different cell lines using gene modification tools such as RNAi and CRISPER-cas9 (Chen et al., 2017). We overlap essential genes in that database with the nodes having high DP values in the *Fibroblast Signaling* network. Some nodes may be proteins that consist of multiple subunits or have multiple isoforms that are encoded by multiple genes. For example, Phospholipase D has two major isoforms, namely PLD 1 and PLD 2. Of these, PLD 1 is found to be essential in one tested cell line grown in GS-9 media (Chen et al., 2017). In such cases, we consider a node as essential if at least one gene (out of all protein coding genes) is listed as essential in the database. The proportion of the essential nodes in top selected nodes having high DP values is compared with the proportion of the essential nodes in the whole network. Using the DAVID tool for pathway enrichment analysis (Huang et al., 2009a,b), the genes associated with high DP nodes are mapped on the KEGG and Biocarta pathways and compared with the total genes in the network as a background. The DAVID tool uses Fisher's exact test to calculate *p*-values. The FDR is computed and a cutoff of 5% is used to correct the multiple comparisons. Furthermore, for annotation clustering the similar terms are clustered together using high classification stringency.
2. **Gene essentiality analysis**
  ***Fibroblast Signaling***: To investigate the essentiality of the nodes with high DP values in the Fibroblast network, we map these nodes with gene essentiality data. Out of 130 nodes in the network, 68 nodes (52%) are essential. To investigate the relationship between essentiality and DP values, we check the distribution of the essential nodes in the top 10, 20, 30, 40, 50, and 60 nodes having high DP values. The essential nodes in these top selected nodes are 70%, 75%, 66%, 60%, 52%, and 53% respectively, as shown in Figure 16A. High proportions of essential nodes are found in the top 10, 20, and 30 nodes. For the top 50 and 60 the proportions are close to the background proportion of essential nodes in the whole network. Among the top 20, a total of 15 nodes (75% of selected nodes) are identified as essential and are listed in Table 2. This proportion is significantly higher than the background proportion of essential nodes in the whole network (*p*-value 0.0306 < 0.05).
  ***T-cell Receptor Signaling***: We investigate the distribution of essential genes in T-cell signaling model. A total of 42 nodes out of 95 (42.2%) are essential. Among the top 10, 20, 30, 40, and 50 nodes having high DP values, 7, 14, 18, 21, and 25 are essential as shown in Figure 16B. We find 70% of nodes as essential in each of the top 10 and top 20 nodes. The proportion of the essential nodes decreases with decreasing DP value. The proportion of the essential nodes in the top 20 nodes having high DP values is significantly higher than that of the background proportion of 42.2% in the whole network (*p*-value 0.0115 < 0.05).
  ***Oxidative Stress Pathway***: Oxidative stress signaling model consists of 18 nodes. Of these, 13 nodes (72.22%) are essential. In the top 5 and top 10 nodes having high DP values, 4 and 7 are essential, respectively. For example, the top hub nodes ROS and AKT are essential.
  ***Cholesterol Regulatory Pathway***: Out of 34 nodes, 7 are essential. The top hub node msREBP is essential in metabolic reprogramming of the effector T-cells (Kidani et al., 2013).
  Thus, nodes having high DP values are enriched with essential genes suggesting that the DP values might be used to predict the gene or protein essentiality.
  We include here a note on how the gene essentiality results relate to the cutoff *L* for the subnetwork size. For example for the *T-cell Receptor Signaling* network shown in Figure 16B, we find 53% essential nodes among the top *L* = 40 nodes having high DP values, in comparison to the 44% essential nodes in the whole network. Similarly, for the *Cholesterol Regulatory Pathway* network a total of 7 essential genes (20%) are found. Of these, 5 nodes are in the top *L* = 22 nodes having high DP. Furthermore, in the case of the *Oxidative Stress Pathway* network, we find 5 essential nodes out of *L* = 6 nodes compared to 13 out of the 18 in whole network. Thus, our chosen cutoff *L* seems to be sufficient for identifying a large fraction of essential nodes. Moreover, the results suggest that even smaller values of the cutoff *L* would allow a significant identification of essential nodes.
3. **Biological pathway analysis**
  ***Fibroblast Signaling***: Further, to investigate the biological processes associated with top DP nodes, we perform pathway analysis of nodes having high DP values (Top 20). We obtain 15 KEGG pathways including signaling pathways such as TNF-alpha signaling, MAPK signaling, and TLR signaling, and pathways associated with diseases such as influenza A infection, viral carcinogenesis, prion diseases, and Epstein-Barr virus infection. The results are shown in Figure 17A. The Erk node is common among 14 out of 15 enriched pathways. Next to this, the Mek node is common among 13 out of 15 enriched KEGG pathways. The EGFR node that has the highest DP value is involved in 5 KEGG pathways. These results suggest that the nodes having high DP values are involved in crucial biological functions, and are also associated with a variety of infections and diseases.
  ***T-cell Receptor Signaling***: Among the top 20 nodes having high DP values, we obtain 13 enriched KEGG pathways as seen in Figure 17B. These enriched pathways include insulin signaling, and pathways involved in diseases such as cancers, long term depression, and alcoholism. The node Raf is common among 12 out of 13 enriched KEGG pathways. The pkb node has the highest DP value in the *T-cell Receptor Signaling* network and is involved in 8 out of 13 enriched KEGG pathways. These results suggest that the nodes having high DP values are involved in crucial biological functions, and also associated with a variety of diseases including cancers.
  ***Oxidative Stress Pathway***: Among the top 5 nodes, the KEGG pathways including renal cell carcinoma, acute myeloid leukemia, prolactin, estrogen, B-cell receptor, and the T-cell receptor are found to be enriched.
  ***Cholesterol Regulatory Pathway***: Among the top 20 nodes no KEGG pathway is found to be enriched.

**Table 2:** Essential genes among the Top 20 nodes having high DP values in the *Fibroblast Signaling* network.

**Fibroblast Nodes (Top 20)**	**Essential Genes (Uniprot ID's)**
ASK1	Q99683
CaM	Q96HY3
Cas	P56945
Cdc42	P60953
EGFR	Q504U8
Erk	P28482, Q8TD08, P27361, Q16659, P31152, Q13164, P53778
Fak	Q05397
IL1_TNFR	P01584, P19438
Mek	Q02750, P36507, P52564, P46734
PKA	P17612, P22694, P22612
PKC	P17252, P05771, P24723, Q05513, Q04759, Q02156, Q05655, P41743
PP2A	P67775
Rho	P08100
Src	P12931
Trafs	Q9BUZ4, Q9Y4K3

## 5. Discussion

The biological function analysis of the nodes having high DP values (hubs) in the *Fibroblast Signaling, T-cell Receptor Signaling, Oxidative Stress Pathway*, and *Cholesterol Regulatory Pathway* networks suggest that the majority of nodes are essential and also involved in crucial biological functions. The proportion of the essential nodes among nodes having high DP values (e.g., top 20) in large scale models, i.e., *Fibroblast Signaling* (130 nodes) and *T-cell Receptor Signaling* (94 nodes) is significantly higher than that of the total essential nodes in the whole network. On the other hand, the comparatively small models *Oxidative Stress Pathway* and *Cholesterol Regulatory Pathway* models also exhibit their hub nodes as essential. The biological pathway analysis of top hub nodes shows that these are involved in important disease pathways.

To have a better understanding of the meaning of the subnetworks of hubs in the more general context of the whole networks, we provide further insight into the biological roles of some of the top hubs in each of the four networks.

The *Fibroblast Signaling* network is a generic network that consists of several major signaling pathways including the Epidermal Growth Factor Receptor (EGFR), the G-protein coupled receptor, and the integrin signaling pathway (Puniya et al., 2016). In the *Fibroblast Signaling* network the nodes with the highest DP values e.g., EGFR, Apoptosis signal-regulationg kinase 1 (ASK1), Erk, Focal adhesion kinase (Fak), Cellular apoptosis susceptibility (Cas) protein, Calmodulin (CaM), or Mek have critical functions in the protein kinase activity, the regulation of protein kinase activity, and the cell proliferation and apoptosis. For example, the hub node EGFR is found to be essential for several biological functions, such as in Toll-like Receptor 3 signaling in human and mouse cell types, including fibroblast, dendritic cells, and macrophages (Yamashita et al., 2012).

The *T-cell Receptor Signaling* network comprises the T-cell receptor, its co-receptors and the transcription factors involved in T-cell activation and function (Saez-Rodriguez et al., 2007). In this network, the nodes with the highest DP values include Protein Kinase B (pkb), Linker of Activated T-cells (Lat), Fyn, Zap70, and the tyrosine kinase (lckp1), that have important roles in the T-cell receptor signaling. The hub node Zap70 is a tyrosine kinase that is essential for the adaptive immune response (Wang et al., 2010). Furthermore, the protein associated with the Lat node is phosphorylated by Zap70 following the T-cell receptor activation (Paz et al., 2001). The other nodes, i.e., pkb, Fyn, and lckp1, are tyrosine kinases involved in cell growth and proliferation (Safran et al., 2010).

The *Oxidative Stress Pathway* network comprises the oxidative stress and PI3K/Akt signaling. In this network, the nodes reactive oxygen species (ROS), Akt and the Anti-oxidant response element (ARE) have the highest DP values. ROS plays an important role in the maintenance of the redox balance. Increased levels of ROS causes macromolecules and cell organelle damage, and triggers the cell apoptosis (Redza-Dutordoir and Averill-Bates, 2016). On the other hand Akt is a positive regulator of cell proliferation.

The *Cholesterol Regulatory Pathway* network consists of reactions involved in cholesterol biosynthesis and its regulation by Sterol regulatory element-binding proteins (SERBPs). The nodes with the highest DP values include mSREBP, Statins, and Acetyl-CoA, and have important roles in regulation. The node mSREBP is a transcription activator involved in the lipid biosynthesis pathway (Shimano, 2001). The Statins are inhibitors of cholesterol biosynthesis. The Acetyl-CoA is a central metabolite and a substrate for cholesterol biosynthesis.

We also point out that many essential nodes may tend to have a large number of outputs, and since the DP is a summation of MI values over all possible outputs, there is a natural correlation between higher DP values and larger number of outputs, as noted in Matache and Matache (2016) and as seen in Figure 11. However, the DP method can identify essential nodes with both large and small number of outputs.

For example, in the *Fibroblast Signaling* network, the top DP node is *EGFR* having 13 outputs. It is identified as an essential node. In Matache and Matache (2016) it is specified that mutations of the *EGFR* are known to be related to lung cancer, interfering with the signaling pathways within the cell triggered to promote cell growth and division (proliferation) and cell survival. The second node in the order of DP is *ASK1*, also an essential node. This node has only 4 outputs and plays important roles in many stress-related diseases, including cancer, diabetes, cardiovascular, and neurodegenerative diseases. The third is the proto-oncogene tyrosine-protein kinase (*Src*), identified as essential. This node is involved in the control of many functions, including cell adhesion, growth, movement and differentiation, and has 30 outputs. Although the fourth node Phosphatidylinositol (3,4,5)-trisphosphate (*PIP3_345*) has 17 outputs, it is not considered essential in the OGEE database (Chen et al., 2012). In fact, among the top 20% of nodes with large DP values, we identify as essential 80% of those with large (≥6) number of outputs and 50% of those with small (≤ 5) number of outputs. The average number of outputs is 4.3 and the maximum is 30 in the *Fibroblast Signaling* network.

A fairly similar situation occurs for the *T-cell Receptor Signaling* network. This suggests that future studies will need to look at further correlations between essentiality and DP values.

We note here that the codes used for the work in this paper are available upon request.

## 6. Conclusions

Our results suggest that DP can serve as a useful tool to identify a subset of relevant nodes in the network that offer the most information gain and whose knowledge reduces the entropy of the whole network significantly. Moreover, many of the nodes with top DP values are identified as biologically essential.

Several directions for further research include extending the data to other networks to increase our samples for the statistical analysis, as well as identifying some network properties or attributes that are potentially correlated with the DP values, such as average bias of the outputs of nodes, canalizing depth, clustering coefficients, or feedback loop information. Moreover, most biological networks have a very large maximal strongly connected component called the “core” (Steinway et al., 2015; Gan and Albert, 2016). On the other hand, it has been shown that disrupting nodes that do not belong to the core may have a significant impact on the network (Palumbo et al., 2005, 2007). More precisely, essential mutations corresponding to enzymes whose elimination has lethal effects on a metabolic network, tend to have a peripheral position and are seldom located in highly connected components of the network. It would be of interest to know how the DP values in the core differ from those not in the core to possibly unravel further correlations.

Another topic for further research is to perform actual network reduction to its top DP nodes and compare the dynamics of the subnetwork to the dynamics of the entire network to explore further the ability of the subnetwork to capture important dynamical aspects of the whole network, such as preservation of attractors. For instance, it would be of interest to explore the Java software GINsim (Naldi et al., 2009a) to actually perform the network reduction and use it to analyze dynamics of the various models found in Cell Collective. This endeavor will require a suitable algorithm for eliminating the edges or connections linking the nodes of the chosen subnetwork to the eliminated nodes.

Some more theoretical approaches would be to study the impact of network reduction for homogeneous networks (that is, networks in which all nodes obey a certain type of Boolean function) to set some baseline dynamical behavior to be used for comparison with more realistic network models.

## Author contributions

TP developed some of the computer codes needed for data collection from the Cell Collective, and he performed most of the simulations needed to generate the data related to the DP values and the subnetwork size for all networks under discussion. He also wrote some of the related parts of the manuscript. BP applied the selected methods for analyzing the biological relevance of the most determinative nodes and wrote the related parts of the manuscript. TH selected the most suitable methods for analyzing the biological relevance of the most determinative nodes, and provided support with network selection, accuracy of approach, and biological information. He wrote the related parts of the manuscript and formatted it for submission. MM devised the mathematical method for finding the subnetwork size, the computer codes for calculations of the DP values and the subnetwork size, and she performed the statistical analysis. She wrote the related parts of the manuscript and formatted it for submission. All authors have contributed to the revision of the manuscript and have agreed on the final draft.

### Conflict of interest statement

TH has served as a shareholder and/or has consulted for Discovery Collective, Inc. The remaining authors declare that the research was conducted in the absence of any commercial or financial relationships that could be construed as a potential conflict of interest.
